# The *Schistosoma mansoni* genome encodes thousands of long non-coding RNAs predicted to be functional at different parasite life-cycle stages

**DOI:** 10.1038/s41598-017-10853-6

**Published:** 2017-09-05

**Authors:** Elton J. R. Vasconcelos, Lucas F. daSilva, David S. Pires, Guilherme M. Lavezzo, Adriana S. A. Pereira, Murilo S. Amaral, Sergio Verjovski-Almeida

**Affiliations:** 1 Laboratório de Expressão Gênica em Eucariotos, Instituto Butantan, 05503-900 Sao Paulo, SP Brazil; 2 Departamento de Bioquímica, Instituto de Química, Universidade de São Paulo, 05508-900 Sao Paulo, SP Brazil; 3 Present Address: College of Veterinary Medicine, Western University of Health Sciences, Pomona, CA USA

## Abstract

Next Generation Sequencing (NGS) strategies, like RNA-Seq, have revealed the transcription of a wide variety of long non-coding RNAs (lncRNAs) in the genomes of several organisms. In the present work we assessed the lncRNAs complement of *Schistosoma mansoni*, the blood fluke that causes schistosomiasis, ranked among the most prevalent parasitic diseases worldwide. We focused on the long intergenic/intervening ncRNAs (lincRNAs), hidden within the large amount of information obtained through RNA-Seq in *S. mansoni* (88 libraries). Our computational pipeline identified 7029 canonically-spliced putative lincRNA genes on 2596 genomic loci (at an average 2.7 isoforms per lincRNA locus), as well as 402 spliced lncRNAs that are antisense to protein-coding (PC) genes. Hundreds of lincRNAs showed traits for being functional, such as the presence of epigenetic marks at their transcription start sites, evolutionary conservation among other schistosome species and differential expression across five different life-cycle stages of the parasite. Real-time qPCR has confirmed the differential life-cycle stage expression of a set of selected lincRNAs. We have built PC gene and lincRNA co-expression networks, unraveling key biological processes where lincRNAs might be involved during parasite development. This is the first report of a large-scale identification and structural annotation of lncRNAs in the *S. mansoni* genome.

## Introduction

Over the past ten years, the advent of Next Generation Sequencing (NGS) strategies like RNA-Seq have revealed the transcription of a wide variety of non-protein-coding RNAs (ncRNAs) in the genomes of eukaryote organisms. Early on this age, it had become quite evident that life in the eukaryotic cells is orchestrated by complex regulatory networks in which ncRNA molecules appear to be the most numerous and main signaling effectors on many vital reactions^1^. Within this large class of ncRNA genes, long non-coding RNAs (lncRNAs) have been conventionally defined as being over 200 nt-long transcripts that have no protein-coding (PC) potential^2^. LncRNAs seem to display a number of regulatory functions and many of them now have a well-characterized role as epigenetic modulators^3–5^.

There are several subtypes of lncRNAs, which are classified by their architecture and/or location onto the genome rather than by their function. The following are the terminologies established for the already known lncRNAs’ subtypes: (*i*) antisense lncRNAs, which overlap the exons of PC genes on the opposite strand; (*ii*) sense intronic lncRNAs, which reside exclusively into PC gene introns; (*iii*) large intergenic/intervening non-coding RNAs (lincRNAs), which do not overlap PC genes and may act either in *cis*, like enhancer RNAs (eRNAs), or in *trans*, by binding to chromatin modifier protein complexes^2, 3, 6, 7^.

Despite sharing similar general traits with their PC gene counterparts such as being transcribed by RNA polymerase II, following the same chromatin state rules, often 5′-capped, spliced and polyadenylated^2^, it is known in vertebrates that lncRNAs exhibit several peculiar and distinct features when compared with mRNAs, such as: (*i*) rapid evolution across species from the same clade^8, 9^; (*ii*) both low and transient expression, being most of the times specimen-, tissue-, and/or cell-specific^6, 7, 10–12^; (*iii*) reduced splicing efficiency^10^; (*iv*) high propensity to *cis-*regulatory action immediately following or during their own transcription^2, 13^; (*v*) participation in *trans-*acting mechanisms when associated to polycomb repressive complex 2 (PRC2), for instance^3, 14, 15^; (*vi*) formation of RNA-DNA triplex structures that may mediate both *cis-* and *trans-*actions^14, 16–18^; (*vii*) more enriched in the nucleus than in the cytoplasm^6^.

*Schistosoma mansoni* was chosen by us as a target organism to unravel the class of lincRNA genes because of both the lack of lncRNAs annotation on its genome and its global health importance as a neglected tropical disease-causing agent. It is a flatworm endemic in both Africa and South America continents that causes an infectious and parasitic disease known as schistosomiasis that affects, together with other schistosome species, over 250 million people worldwide^19^. Besides its public health impact, this parasite is also an interesting model for studies on the Molecular Parasitology field because of its complex life cycle and, consequently, the drastic gene expression changes it faces during development. From the nearly 12,000 PC genes already mapped onto *Schistosoma spp*. genomes^20–22^, there are about 2,000 stage-specific differentially expressed (DE) ones among *S. mansoni* cercariae (the human infective larval stage), schistosomula (the first stage in a mammal host) and mature mixed adults residing in the mesenteric veins^21^, as well as between separate male and female adult forms of *S. haematobium* and their respective eggs^22^.

Up to date only one single study has been devoted to a large-scale mapping of ncRNA genes in schistosomes^23^. However, the authors have only adopted homology-based annotation through the use of bioinformatics tools in order to screen for structured RNAs on a comparison between *S. mansoni* and *S. japonicum* genomes^23^. No lncRNAs were reported on that investigation. On the other hand, our group has already pointed to the existence of lncRNAs in the *S. mansoni* transcriptome, by using both microarray and low-coverage RNA-Seq approaches^24, 25^.

Recently, several new bioinformatics tools have been developed with the aim of capturing novel lncRNAs mapped to the genomes^26–29^. However, they are mostly geared towards applying the machine learning techniques to the well-curated human and/or mouse transcriptome mapping annotations. For invertebrates, *Caenorhabditis elegans* (a nematode free-living worm) and *Plasmodium falciparum* (a protozoan parasite) have already had their transcriptomes screened for lncRNAs by independent groups who established their own *ad-hoc* pipelines for analyzing RNA-Seq data^30, 31^. They have unraveled ~1,000 transcripts among antisense lncRNAs and lincRNAs for each of the above two organisms.

In the present work, we thoroughly screened the *S. mansoni* genome and transcriptome and we present a novel repertoire of spliced lncRNA genes, with a main focus on the lincRNAs subtype. We first describe our computational pipeline aimed at retrieving lncRNAs from dozens of raw large-scale RNA-Seq samples (either public data available at the NCBI-SRA repository or data generated by us). Subsequently, we report their location and architecture onto the genome, the evidence for being transcribed, and their evolutionary conservation among other species. Finally, we analyze the lincRNAs putative functionality by building co-expression networks that associate them to their PC gene counterparts that are co-expressed across five different parasite developmental stages. This is the first study that provides a holistic systems overview that merges *S. mansoni* lincRNAs content with what is encoded into proteins and raises hypotheses about the lincRNAs functionality through the construction of co-expression networks.

## Results

### A computational pipeline is able to retrieve thousands of lncRNAs from *S. mansoni* RNA-Seq samples

**Figure 1:**
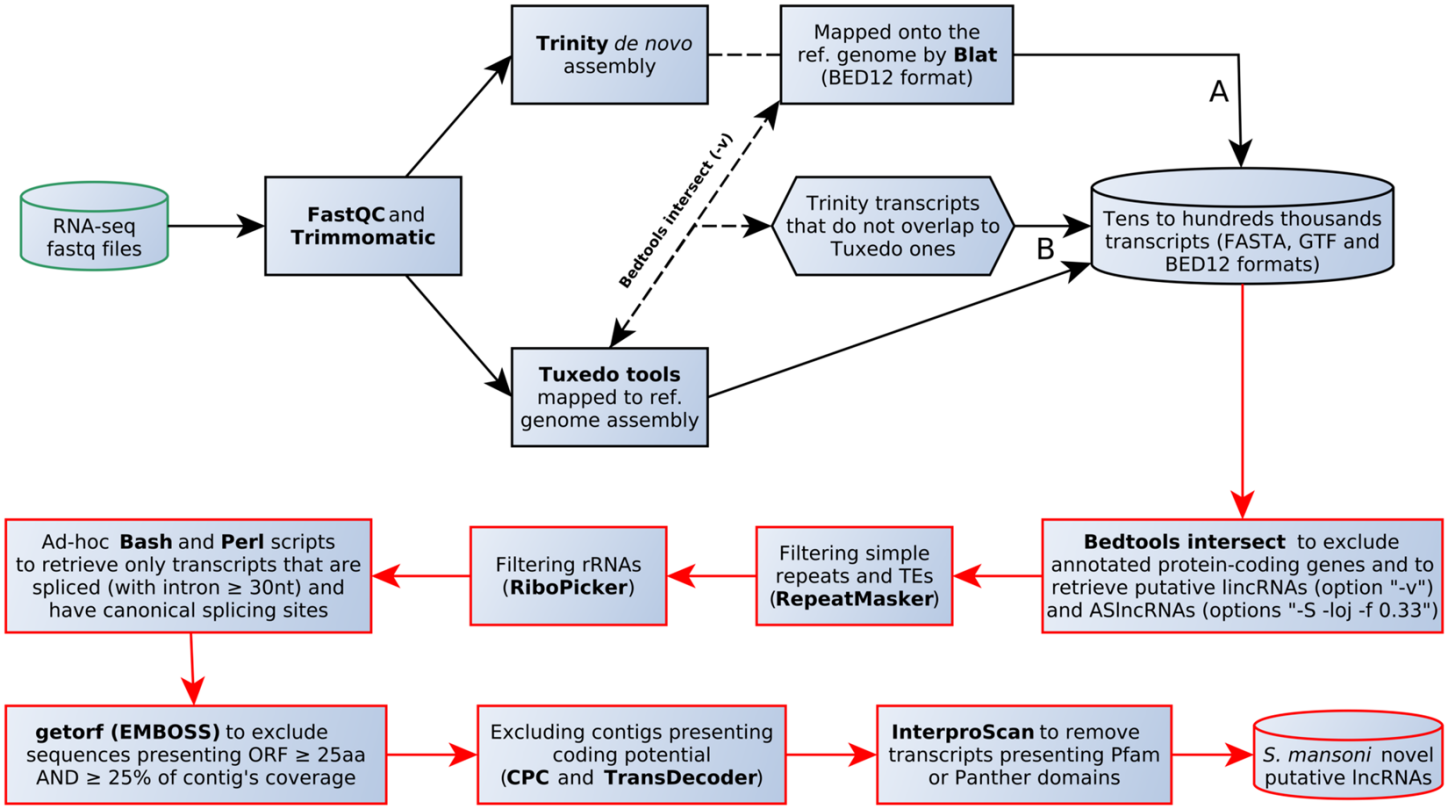
Computational workflow for rescuing and identifying novel putative lncRNAs from *S. mansoni* high-throughput transcriptomic data. Path A depicts the use of a *de novo* assembly method, whereas path B comprises reference-guided assembly. The red section is built in one single PERL script for the automation of tasks. On the bedtools intersect step (first red rectangle), one can choose whether the pipeline retrieves antisense lncRNAs or lincRNAs.

Our *ad-hoc* computational pipeline (Fig. 1) was designed based on two distinct transcriptome assembly approaches: (*i*) a *de novo* method using Trinity^32^ (Fig. 1, path A), and (*ii*) a reference genome/annotation-based method, using Tophat2 (ref. 33), Cufflinks and Cuffmerge^34^ (the Tuxedo suite) (Fig. 1, path B). As suggested by Ulitsky^9^, the use of different assembly algorithms increases the chance of detecting the lncRNAs complement of a cell or organism (more details on the Methods section). Table 1 shows the number of transcripts obtained with each assembly algorithm.

**Table 1:** Summary of *S. mansoni* lincRNAs mining steps and their respective number of filtered transcripts after the execution of the pipeline from Fig. 1.

Filtering Steps	Number of transcripts for each indicated dataset and assembly tool that remain after the indicated filtering step			
	trin_strA*	trin_strB*	trin_sra*	tux_all*
Total assembled transcripts	245,237	230,961	389,266	83,821
Remove annotated protein-coding genes	54,927	48,269	79,684	10,531
Remove repeats	43,132	36,440	61,820	7928
Remove rRNAs	43,123	36,421	61,810	7928
Remove monoexonic transcripts	16,499	13,779	15,324	4677
Remove transcripts with all introns < 30 bp	11,026	9398	7347	4642
Remove transcripts with non-canonical splice sites	7355	6294	2207	4347
Remove transcripts with ORFs ≥ 25 aa (AND ≥ 25% transcript length)	2495	2032	628	2767
Remove transcripts with coding potential (CPC and TransDecoder)	2432	1985	613	2250
Remove transcripts with protein domains (Interproscan)	2407	1956	604	2062
Total *S. mansoni* novel putative lincRNAs (sum of the four datasets)	7029			

*Two different assembly methods (Trinity *de novo* “trin” and Tuxedo tools genome-guided “tux”) and four datasets (strA, strB, sra and all) were used as starting point for lincRNAs identification (see Methods for more details).

We have established a series of more stringent filtering steps in our pipeline (red border rectangles on Fig. 1) compared with the previously described ones for other invertebrates^31^, and we used a greater number of RNA-Seq samples as input (n = 88, see Methods). The first filtering step was the removal of PC-overlapping transcripts when rescuing lincRNAs, or the removal of both intervening and sense PC-overlapping transcripts when rescuing the antisense lncRNAs. As an example, Table 1 shows the number of lincRNA transcripts that remain at this step and at each of the further ones in our pipeline. The subsequent steps included the removal of repeats (low complexity and/or transposable elements), rRNAs, monoexonic transcripts, and spliced transcripts presenting all introns with less than 30 nt each and/or not showing canonical splice donor/acceptor (GT/AG) sites. Further, we removed transcripts presenting ORFs greater than 25 amino acids (aa) and covering 25% or more of the transcript lengths, as well as transcripts assigned as having coding potential probability by either CPC^35^ or TransDecoder^36^ algorithms, and, finally, transcripts containing Pfam and/or PANTHER domains as detected by InterProScan^37^ (see Methods).

**Figure 2:**
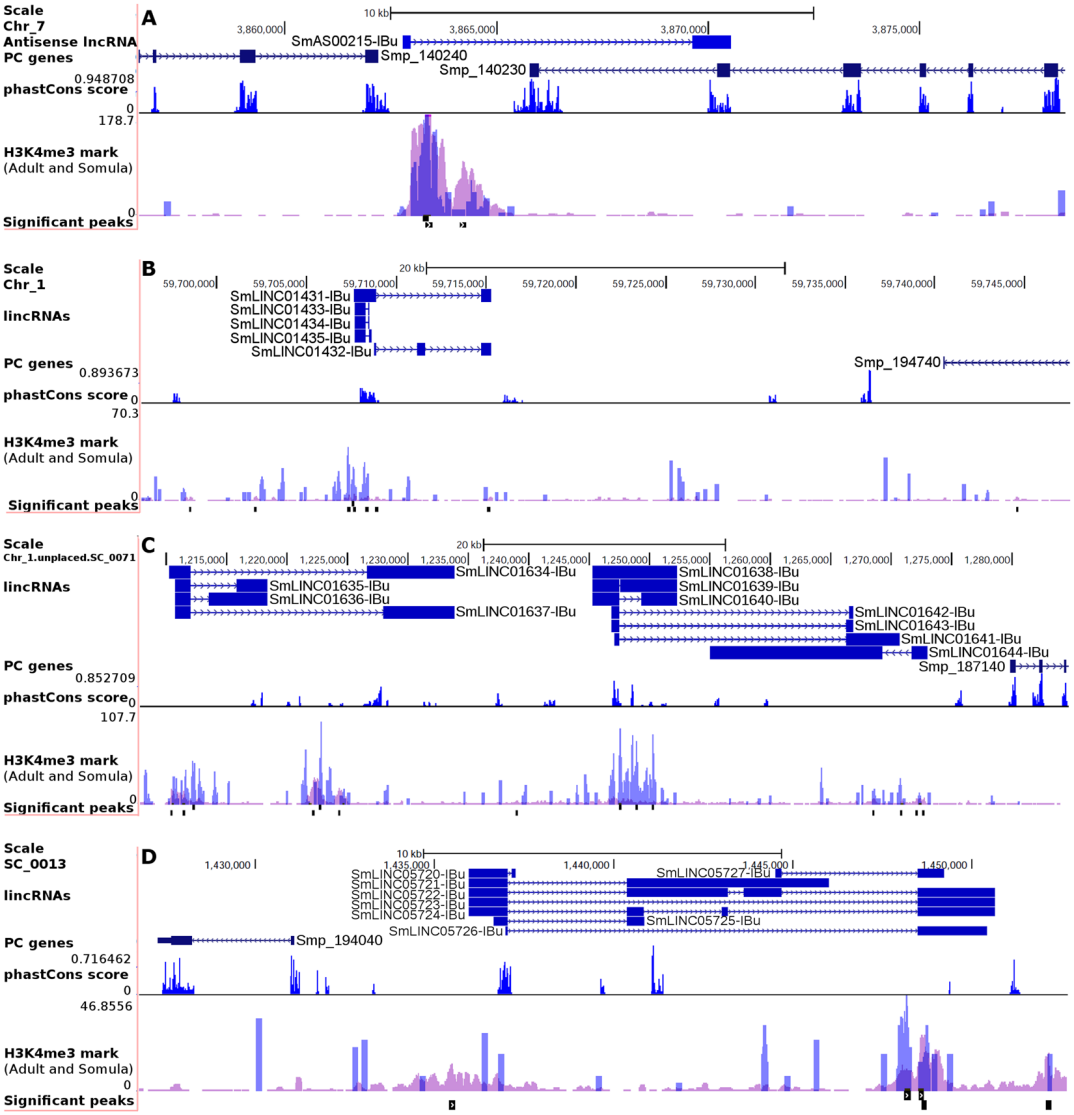
Novel *S. mansoni* spliced lncRNA gene loci. All lncRNA isoforms depicted on the figure display both evolutionary conservation (phastCons score) among other schistosome species (*S. haematobium* and *S. japonicum*) and epigenetic marks (H3K4me3) that correspond to transcription initiation. **(A)** LncRNA antisense to a 78 kDa centrosomal protein gene (Smp_140230). The PC genes’ “tail to tail” orientation indicates that the H3K4me3 marks are exclusive to the lncRNA gene. **(B)** LincRNA locus upstream of a G protein coupled receptor gene (Smp_194740) of which only the last exon is shown. (**C**) LincRNA loci upstream of a cathepsin L proteinase gene (Smp_187140). (**D**) LincRNA loci downstream a hypothetical protein gene (Smp_194040). Both protein-coding and lncRNA gene IDs are searchable on the *S. mansoni* UCSC-like genome browser which our group has deployed (http://schistosoma.usp.br) and previously reported^24^.

At the end of the filtering pipeline we obtained two datasets: one with 7029 lincRNA isoforms (Table 1) located on 2596 genomic loci (at an average of 2.7 lincRNA isoforms per each lincRNA genomic locus), and another dataset with 402 lncRNAs that are antisense to 268 PC genes. The transcript sequences are on the Supplementary File S1. On Supplementary Table S1 we provide the genomic location and architecture of each lncRNA for the two datasets. We decided to focus our analyses on the lincRNAs gene set due to the greater amount retrieved, and also because the lincRNAs arise from PC-independent novel gene loci. For a better visualization of the lincRNA loci and architecture, we have created a track for them on the *S. mansoni* UCSC-like genome browser (http://schistosoma.usp.br), which we recently implemented and reported^24^. On Fig. 2 we selected three lincRNA loci to be viewed as genome browser pictures, where one can see different tracks on each of three different loci, showing evidence for transcription and evolutionary conservation of these novel lncRNA genes. Such evidence will be described further on this section.

Because there is no gold standard *in silico* protocol for detecting lincRNAs in ancient parasite genomes/transcriptomes, we ran a sensitivity test of our pipeline, namely trying to reproduce the list of previously identified lincRNAs from *Plasmodium falciparum* ^30^, and we successfully recovered 53% of the *P. falciparum* lincRNAs. We believe the 53% sensitivity rate reflects the more stringent filtering steps that we have deployed on our pipeline compared with the ones from Broadbent *et al*.^30^. We expect that low levels of false positive lncRNAs detection may be achieved in our *S. mansoni* data analysis due to our stringency choices.

### A great variety of lincRNA transcript isoforms was detected

In order to check whether different assembly tools and datasets used as input in our pipeline (see Table 1) yielded similar transcripts, and to calculate the degree of redundant transcripts obtained, we performed a blastn^38^ alignment of the total 7029 lincRNA sequences against themselves. This all against all comparison has revealed only 15 identical pairwise alignments (100% identity and 100% coverage of both query and subject sequences) encompassing 21 lincRNAs (0.3% redundancy) (Supplementary Table S2). Excluding the requirement of complete identity and coverage, and computing the pairs in which one transcript is completely contained within a longer transcript isoform (i.e. lincRNA queries completely covered by the subject lincRNA with 100% identity and with partial subject coverage) the number of pairs was increased to 281 (4%). This reflects the presence of quite similar isoforms in our assembled transcripts dataset.

Further agreeing with the existence of multiple isoforms of each lincRNA per locus, we found that, keeping the 100% identity threshold but decreasing the requirement of the extent of query coverage (qcov), the all against all comparison has identified approximately similar numbers of lincRNA queries being aligned to the subject at the different qcov ranges: 1422 (90% ≤ qcov < 100%), 1310 (80% ≤ qcov < 90%), 1304 (70% ≤ qcov < 80%), 1327 (60% ≤ qcov < 70%), 1221 (50% ≤ qcov < 60%) (Supplementary Table S2).

In summary, sorting all these lincRNA query lists and eliminating the redundancy, we obtained 4212 (60%) lincRNAs that share 100% identity on at least half of their length with other lincRNAs in the dataset. This result indicates that different transcriptome assembly approaches plus different samples used as input lead to the detection of distinct lincRNAs transcript isoforms. These isoforms mapped to 2596 unique genomic loci, as seen above. Finding different lncRNA sets when using different RNA-Seq datasets and assembly tools was already reported when analyzing sets of different individuals, tissues and even at the single-cell level^8–10, 12^. We therefore decided to keep in our downstream analyses all the 7029 *S. mansoni* putative lincRNAs (hereafter called SmLINCs) as *bona fide* representatives of the complement of expressed *S. mansoni* lincRNAs.

We analyzed the density of lincRNAs mapped to the long, assembled chromosomes and found that the average density was 1.86 lincRNAs per 100 kb of genomic sequence (range 1.31 to 2.30) (Table S3), with no higher density in any particular chromosome. A linear correlation between chromosome size and number of lincRNAs per chromosome was obtained (coefficient of determination R^2^ = 0.97) (Table S3). A higher density of lncRNAs in certain chromosomes was found in vertebrates such as in the mouse, where there is a higher density of lncRNAs in chromosomes 2, 4 and 11 (ref. 39); this pattern was not observed in *S. mansoni*.

### *S. mansoni* putative lincRNAs (SmLINCs) display evidence of being functional genes

We have selected four features to scrutinize our *S. mansoni* putative lincRNAs dataset in the search for evidence of their possible functional role: (*i*) presence of histone H3 lysine 4 trimethylation (H3K4me3) on the transcription start site (TSS) as an epigenetic mark for transcriptional activation; (*ii*) evolutionary conservation among two other schistosomes; (*iii*) differential expression across five parasite developmental stages; and (*iv*) positive or negative expression correlation to their PC gene neighbors.

**Figure 3:**
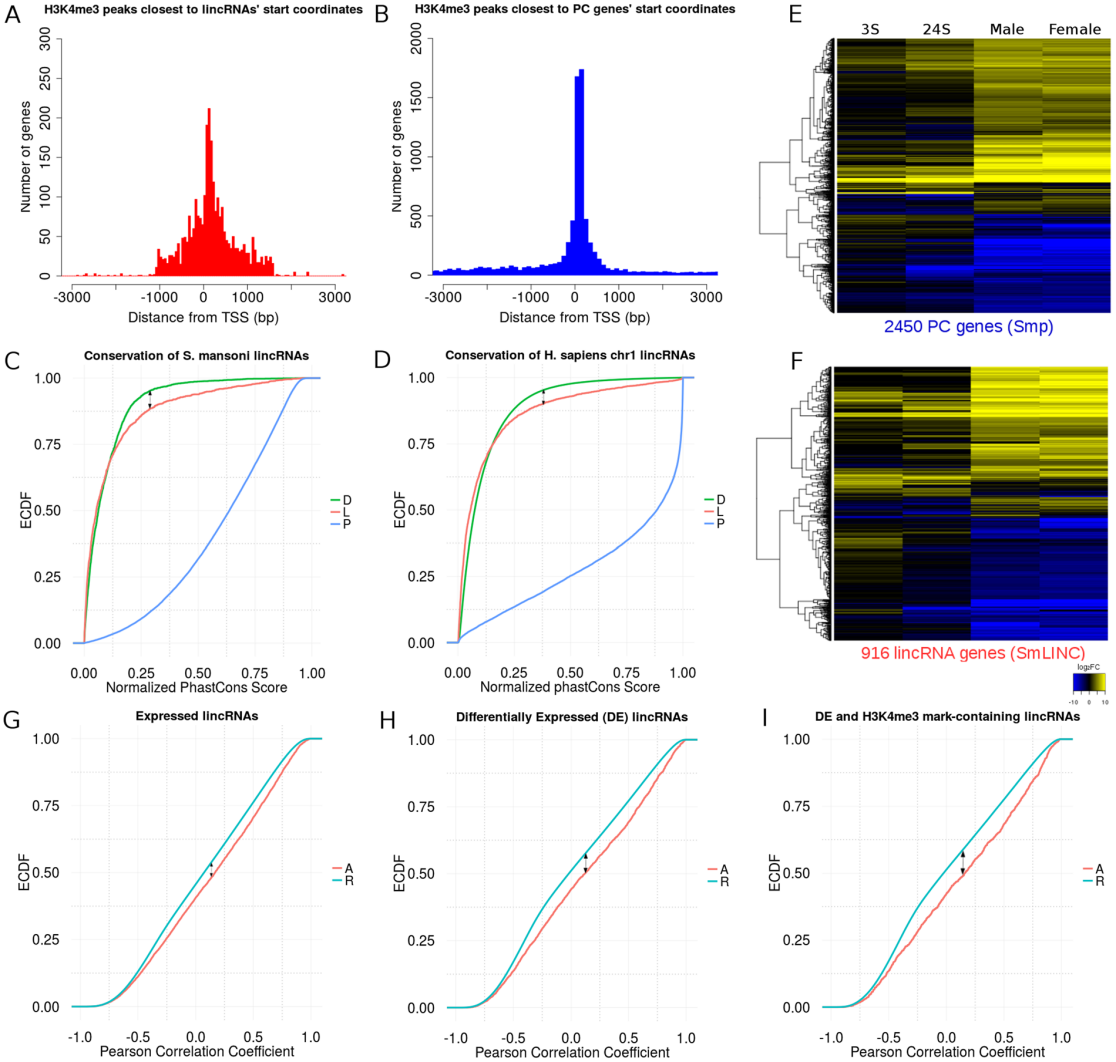
*S. mansoni* lincRNAs display four different traits that may characterize them as functional genes. (**A,B)** transcriptional activation epigenetic mark (H3K4me3) surrounding the TSS on both lincRNAs (red) and PC genes (blue). **(C)** An empirical cumulative distribution function (ECDF) showed that lincRNAs’ (red) evolutionary conservation scores (phastCons) among three schistosome species are significantly different from genomic deserts (green) (arrow: one-sided KS-test p-value = 4.25e-06) and from PC genes (blue). **(D)** As a control, lincRNAs from human chromosome 1 (red) were compared with genomics deserts (green) and with PC genes (blue) from the same chromosome, and they display a phastCons score (comparison among 20 mammals provided by UCSC Table Browser for human genome) ECDF pattern similar to the one observed for *S. mansoni* (arrow: one-sided KS-test p-value = 2.67e-07). **(E,F)** Heatmap of 2450 PC genes (**E**) and 916 lincRNA transcripts (**F**) that were detected as differentially expressed with one-way ANOVA-like analysis comparing RNA-seq samples from somula 3 h (3S), 24 h (24S) and adults (male and female) against cercariae (the average expression of biological triplicates for each life cycle stage was used, and an adjusted p-value threshold of 0.01 was employed). For each gene (lines), the expression log-ratio between the indicated life cycle stage and cercariae was obtained (columns), and it was colored according to the scale indicated at the bottom of the panel F. **(G**–**I)** LincRNAs tend to be co-expressed with their PC gene neighbors (A curves, red) rather than with a set of 1000 randomly picked PC genes (R curves, cyan) during parasite development (same five life cycle stages assessed on E and F panels). Pearson correlation values’ ECDF for 1,572 expressed lincRNAs (TPM ≥ 1) that showed either *r* > 0.5 or *r* < −0.5 with their actual PC gene neighbors (**G**), for 893 differentially expressed lincRNAs (TPM ≥ 1) correlated with their actual PC gene neighbors (**H**) and for 402 DE lincRNAs (TPM ≥ 1) that also have H3K4me3 marks at their TSSs and are correlated with their actual PC gene neighbors (**I**) (arrows: KS-test p-value < 1e-12 for each of the three distributions).

H3K4me3 is a very well studied epigenetic mark present on the promoter region of actively transcribed genes^40^. Moreover, it is already known that such mark is not exclusive to PC genes, being also present on active lncRNA loci^2^. Therefore, we screened the lincRNAs’ TSS surrounding regions for the presence of significant H3K4me3 peaks in the ChIP-Seq data for both somula and adult forms of the parasite (publicly available data on SRA-NCBI^41, 42^). Forty percent of SmLINC (2878/7029) showed the H3K4me3 mark on their TSS surrounding regions. In addition, the pattern of distance between the lincRNAs TSS and the H3K4me3 closest peak (Fig. 3A) was quite similar to the pattern observed for PC genes (Fig. 3B).

Regarding the evolutionary conservation, we computed the phastCons scores^43^ assigned at the single-base level, after whole genomes multiple alignments had been obtained with the MULTIZ-TBA package of tools^44^. As already shown for the human genome^45^, phastCons scores are able to distinguish between features of high selective constraint (PC genes) and the ones of weak conservation (lncRNAs, ancestral repeats and desert regions), and even within the latter group it is still possible to differentiate genomic elements by their phastCons profile. After aligning three schistosomes’ genomes (*S. mansoni*, *S. haematobium* and *S. japonicum*), we obtained phastCons scores for about 56.9 Mb of genome sequence (see Methods), on which 3453 SmLINC were mapped and presented scored/conserved bases on at least one of their exons.

Table 2 presents an overview of three different *S. mansoni* genomic features (PC genes, lincRNAs and desert regions with neither annotation nor transcript evidence) and their phastCons evolutionary conservation scores’ content. As one can see by this rough summary, lincRNAs are more similar to desert regions than to PC genes, corroborating what was already seen in both human and mouse studies^6, 46^. By performing an empirical cumulative distribution function (ECDF) analysis of normalized phastCons scores per block together with a Kolmogorov-Smirnov (KS) test, we were able to distinguish a significantly different evolutionary conservation between SmLINC and desert regions (800 bp) in the *S. mansoni* genome, being the former higher than the latter (KS one-sided p-value = 4.2e-06, Fig. 3C). As a comparison, we calculated the phastCons scores for human chr1 annotated lincRNAs compared with desert regions (400 bp) on the same chromosome, using the 20 mammals phastCons scored bases provided by the UCSC genome browser (https://genome.ucsc.edu) for the human hg38 genome sequence assembly. Similar ECDF profiles were obtained (Fig. 3D, KS one-sided p-value = 2.6e-07). These results are indicative that almost half of the SmLINC reported herein (3453/7029) are under some sort of selective evolutionary pressure in the *Schistosoma* genus.

**Table 2:** Summary of conserved features identified by phastCons on the *S. mansoni* genome.

Genomic Features	# blocks (or exons for gene features)	# phastCons score-containing bases	# phastCons score-containing blocks	# blocks with no score
Protein-coding	69,809	14,247,623	57,974 (83%)	11,835 (17%)
lincRNAs	6,246	559,217	1,567 (25%)	4,679 (75%)
Desert regions	4,134	842,215	1,336 (32.3%)	2,798 (67.7%)
Deserts 800 bp	14,838	842,215	2,503 (16.8%)	12,335 (83.2%)

The scores are related to conservation on three genomes (*S. mansoni, S. haematobium* and *S. japonicum*). Protein-coding represents 11,844 PC genes (Smps) annotated from the latest genome version. LincRNAs are the 7029 assembled transcripts from the current study that do not overlap to Smps. Desert regions are the *S. mansoni* genomic loci with neither gene annotation nor transcript signals from the 88 RNA-Seq samples analyzed herein. These desert regions were split onto 800 bp-long blocks to mimic the mean length of lincRNAs’ exons.

Differential expression along parasite development is another important trait that may characterize lincRNAs as transcripts that are required during the life cycle. We performed a one-way ANOVA-like analysis comparing somula (both 3 h and 24 h) and adult worms (male and female) against cercariae (see Methods for details). First, we identified 2450 differentially expressed (DE) PC genes (Smps) (Fig. 3E), a number close to what was identified by others comparing several developmental stages on both *S. mansoni* ^21^ and *S. haematobium* ^22^. Next, 916 SmLINCs were identified as DE on at least one developmental stage (p-value < 0.01) (Fig. 3F). The complete list of DE genes and their respective log_2_(fold-change), p-value and adjusted p-value are provided as a spreadsheet on Supplementary Table S4. We observed a lower frequency of DE lincRNAs (916/7029 = 13%) compared with DE PC genes (2450/11, 844 = 20.7%). We believe that we might have missed some lowly expressed lincRNAs on our final DE list (having TPM < 1 on all analyzed life cycle stages), since lncRNAs have lower expression levels compared with PCs (Supplementary Fig. S1) and because we have established for the ANOVA-like DE analysis a cutoff TPM ≥ 1 on at least one life cycle stage (three replicates). Similar to what can be seen for DE PC genes (Fig. 3E), DE lincRNAs also display stage-specific up-regulation patterns (Fig. 3F): adult-specific up-regulated genes can be seen on the heatmap’s top half, some somula-specific up-regulated lincRNAs are depicted at the middle portion of the heatmap, and cercariae up-regulated lincRNAs (i.e., somula and adults down-regulated lincRNAs) are observed at the bottom half of the heatmap (Fig. 3F). This result suggests that the lincRNA genes described herein might be important for the parasite development.

The fourth and last evidence sought by us was whether there is an expression correlation between SmLINCs and their PC gene neighbors in the genome, in comparison with control pairs of the SmLINCs and randomly-selected PC genes. First, we picked four PC gene neighbors (the first two upstream and two downstream) for each SmLINC (when it was possible, see below) and computed the pairwise (SmLINC-PC gene) Pearson expression correlation coefficient (*r*) (using TPM expression values for each gene from the same five developmental stages described above for the DE one-way ANOVA-like analysis, Fig. 3E,F). Next, we compared the expression correlation coefficients (*r*) against the ones from a negative control consisting of 1,000 times bootstrapped random PC genes paired to the same SmLINC set.

We started the analysis with 2039 expressed lincRNAs that had TPM ≥ 1 (on all three replicates of at least one life cycle stage), yielding 7269 SmLINC-neighbor_PC gene pairs (some lincRNAs may not have four PC gene neighbors because they can either be located on a scaffold bearing quite few (or no) PC genes or be at the extremities of assembled chromosomes/scaffolds). After comparing the ECDF profiles of all *r-*values computed for the actual 7269 SmLINC-neighbor_PC gene pairs against a negative control consisting of *r-*values for 7,269,000 pairs of SmLINC-randomly_selected_Smps, we observed a significant difference on the two distribution curves (KS one-sided p-value = 5.6e-25, Fig. 3G). It is noteworthy that 1572/2039 SmLINC-neighbor_PC gene pairs (77%) showed significant expression correlation (*r*) p-values < 0.05, whereas in the negative control only 2,547,966/7,269,000 SmLINC-randomly_selected_Smp pairs (35%) had *r* p-values < 0.05.

Next, we reduced the set of lincRNAs in the analysis by keeping only the 893 lincRNAs that are significantly differentially expressed (DE) across the stages within the set of 2039 lincRNAs with TPM ≥ 1. For these 893 DE lincRNAs there were 3167 SmLINC-neighbor_ PC gene pairs, which were compared with 3,167,000 SmLINC-randomly_selected_Smp bootstrapped pairs. The difference between their ECDF curves was also significant (KS one-sided p-value = 6.8e-20, Fig. 3H) and notoriously greater than the difference in the previous ECDF plot (Fig. 3G). Seven hundred thirty two out of the 893 SmLINC-neighbor_PC gene pairs (81.9%) showed significant expression correlation (*r*) p-values (<0.05), whereas 1,206,153/3,167,000 SmLINC-randomly_selected_Smp pairs (38%) had the same correlation significance.

Finally, filtering the 2039 SmLINCs by both being DE and having the H3K4me3 mark at their TSS-surrounding genomic region, we obtained 402 lincRNAs and 1406 SmLINC-neighbor_PC gene pairs. Their ECDF plot showed the greatest difference between the curves (KS one-sided p-value = 1.5e-13, Fig. 3I); in this set, a total of 325/402 SmLINC-neighbor_PC gene pairs (80.8%) had expression correlation (*r*) p-values < 0.05, while this same feature was seen for only 532,925/1,406,000 random pairs (37.3%) in the negative control.

The above results indicate that it is possible that SmLINCs may play a *cis*-acting role regulating their PC gene neighbors, as already known for vertebrates^2, 5^. In addition, as we decreased the lincRNAs’ set by keeping the ones displaying more evidence for being active, we observed an increase in the difference between the correlation of actual neighbors versus random pairs (Fig. 3GHI), suggesting that a set of lincRNAs is more prone to show a co-expression pattern with their PC gene neighbors.

**Figure 4:**
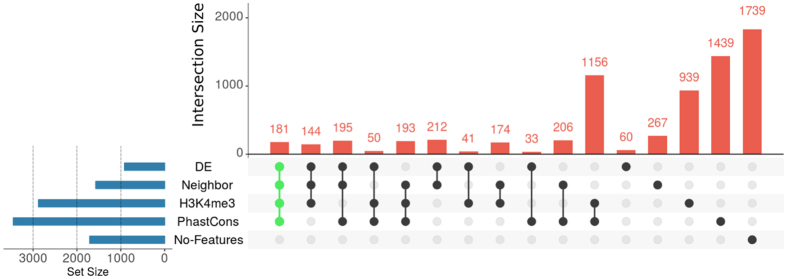
Hundreds of SmLINCs share two or more traits that may characterize them as functional genes. The UpSet intersection diagram shows the number of lincRNAs that were detected as being differentially expressed across five life cycle stages (DE), as having their expression correlated with the expression of protein-coding gene neighbors (Neighbor), as having histone active transcription marks at their TSSs (H3K4me3) and as being conserved among *Schistosoma spp*. (PhastCons). The 181 lincRNAs that share all traits were selected for further investigation using systems approaches.

An intersection diagram displaying the number of *S. mansoni* lincRNAs having from one to four of the analyzed traits that may characterize them as functional genes was obtained for the 7029 lincRNAs (Fig. 4), showing that only 1739 lincRNAs (25%) could not be associated with these traits. We decided to focus our downstream analyses on the main intersection group of 181 lincRNA genes (Fig. 4). The genes that were displayed as genome browser images on Fig. 2 are part of that main intersection set. The complete list of the 181 robust SmLINCs is provided as supplementary file (Supplementary File S2). Each of them can be accessed through our online genome browser (http://schistosoma.usp.br).

### RT-qPCR assays confirm the SmLINCs’ differential expression across distinct developmental stages

Sixteen out of the 181 robust SmLINCs’ set from the UpSet intersection diagram on Fig. 4 were selected for individual assessment of their expression levels on cercariae (C), somula 3 h (3S) and somula 24 h (24S) after mechanical transformation, male and female stages/forms. First, in order to confirm that the RNA samples herein used for RT-qPCR contained previously known stage-specific/enriched transcripts, we measured the expression levels of six protein-coding genes as positive controls (Supplementary Fig. S2). In the RT-qPCR assays (Supplementary Fig. S2), all six selected control PC transcripts corroborated the up-regulation pattern at the life cycle stage that had already been reported in the literature: Smp_044250 (Metalloprotease), shown as up-regulated in cercariae compared with somula^47^; Smp_033040 (Lactate dehydrogenase), up-regulated in somula compared with cercariae^47^; Smp_126730-5HTR and Smp_145140-WNT5, assessed as up-regulated in adult male compared with female^24^, Smp_000390-Trematode Eggshell and Smp_000430-EggShell, up-regulated in adult female compared with male^24^.

**Figure 5:**
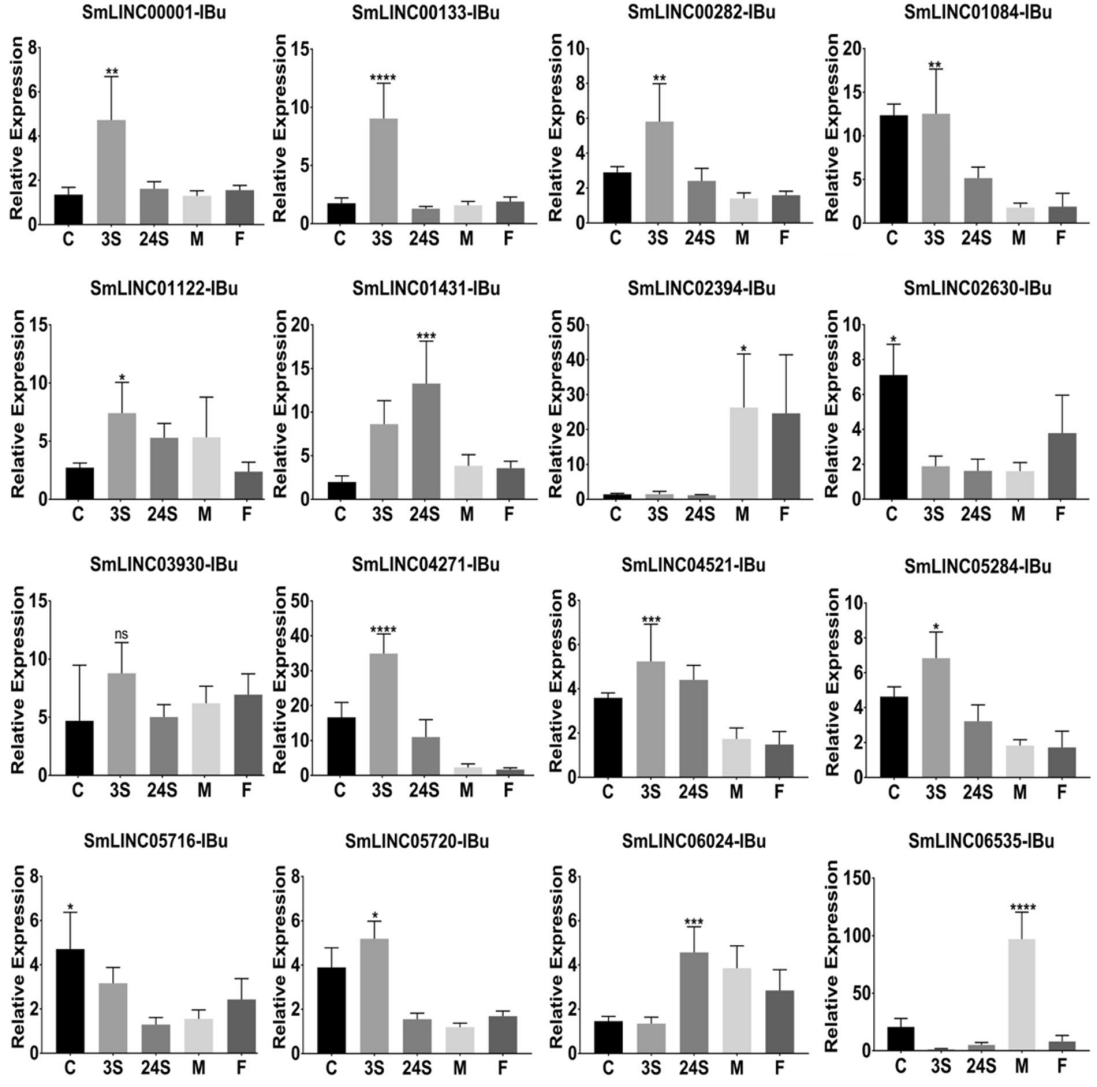
Confirmation by RT-qPCR of the differential expression of selected SmLincRNAs across the parasite life cycle stages. Sixteen SmLincRNAs were selected for validation by RT-qPCR at the parasite stages, namely cercariae (**C**), schistosomula after 3 hours of mechanical transformation (3S), schistosomula after 24 hours of mechanical transformation (24S), adult male and female (x axis). All sixteen lincRNA loci are among the 181 lincRNAs loci shown at the main intersection in Fig. 4. For each lincRNA plot, the individual sample with the lowest normalized expression value across all stages was chosen and arbitrarily set to 1. The expression values of the same lincRNA for all the other samples are represented as the relative expression compared with the lowest one (y axis). Bars represent standard deviation of the mean from four biological replicates for each stage. Three technical replicates were performed for each of the four biological replicates per stage. The Smp_092920 was used for internal normalization as the reference gene among the parasite stages (see Methods). The ANOVA Tukey test was used to calculate the statistical significance of the expression differences among the parasite forms (*p-value ≤ 0.05; **p-value ≤ 0.01; ***p-value ≤ 0.001; ****p-value ≤ 0.0001). For clarity purposes, it is shown only the highest p-value representation for the stage in which the lincRNA was detected with the highest expression.

We then performed RT-qPCR to assess the expression levels of sixteen selected lincRNAs across the five *S. mansoni* stages studied. The primers used are listed on Supplementary Table S5. It was possible to detect differential expression of lincRNAs on all the four life stages: two more highly expressed in cercariae (SmLINC02630-IBu and SmLINC05716-IBu), ten in somula 3 h (SmLINC00001-IBu, SmLINC00133-IBu, SmLINC00282-IBu, SmLINC01084-IBu, SmLINC01122-IBu, SmLINC03930-IBu, SmLINC04271-IBu, SmLINC04521-IBu, SmLINC05284-IBu and SmLINC05720-IBu), two more highly expressed in somula 24 h (SmLINC01431-IBu and SmLINC06024-IBu) and two in male adults (SmLINC02394-IBu and SmLINC06535-IBu) (Fig. 5). Half of the genes showed an expression pattern concordant to those measured by RNA-Seq analysis (SmLINC02630-IBu, SmLINC00133-IBu, SmLINC00282-IBu, SmLINC03930-IBu, SmLINC04271-IBu, SmLINC05284-IBu, SmLINC05720-IBu and SmLINC02394-IBu). It is noteworthy that some SmLINCs from the other half, for which the qPCR did not corroborate the RNA-Seq stage of higher expression, had very low RNA-Seq read counts on all stages (SmLINC01084-IBu, SmLINC01122-IBu and SmLINC01431), which in turn may diminish the detection of differential expression of some lincRNAs by the high-throughput approach.

### Co-expression networks (lincRNAs-PC gene) raise hypotheses about lincRNAs’ functionality

**Figure 6:**
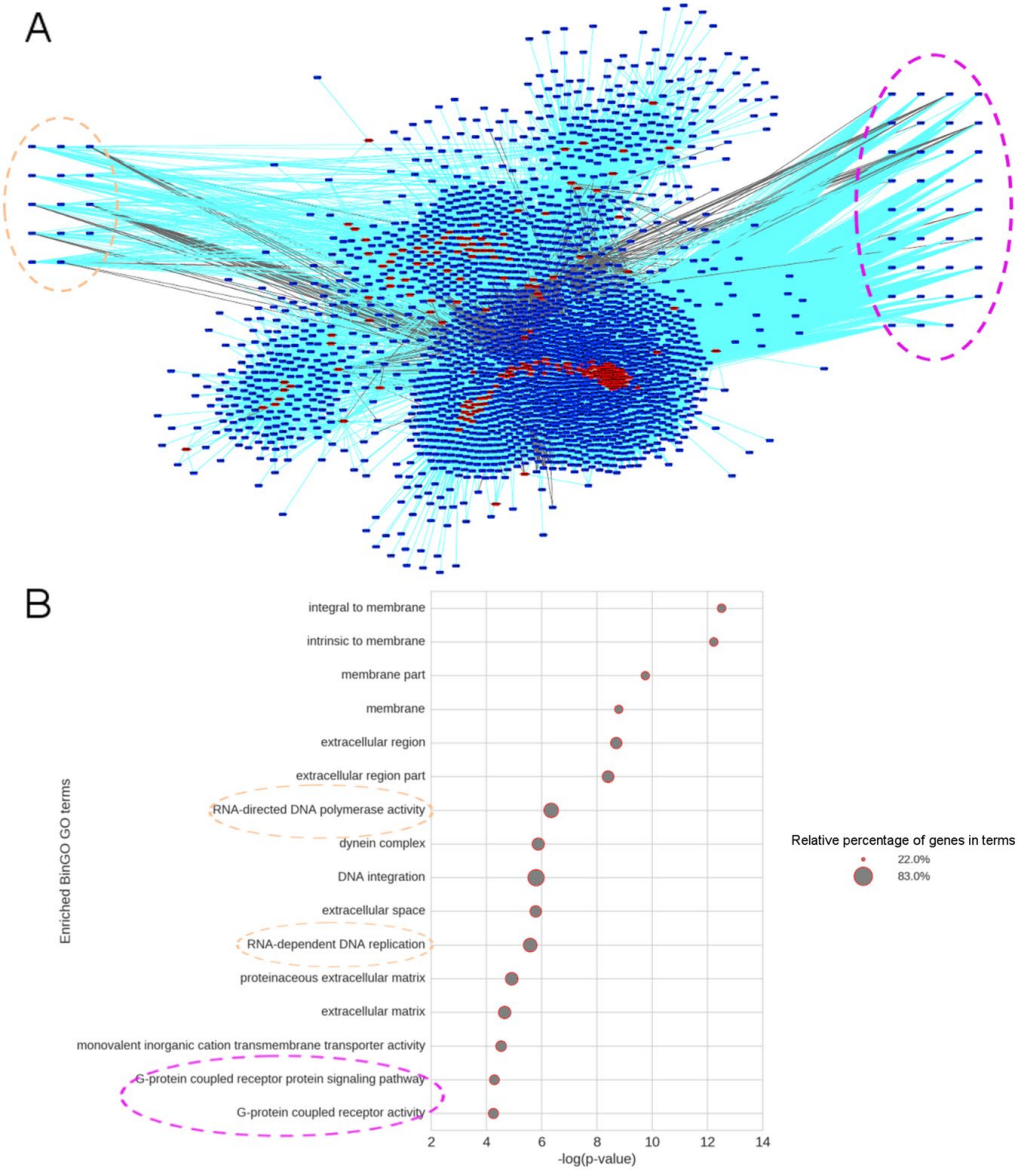
Co-expression gene network (lincRNA-PC gene) pinpoints the processes in which lincRNAs may act. (**A)** The 181 robust lincRNAs (red nodes; lincRNAs from the main intersection in the UpSet Intersection Diagram on Fig. 4) were used as bait for catching 2359 PC genes (blue nodes) whose expression levels across five developmental stages are either positively (*r* ≥ 0.8, cyan edges) or negatively (*r* ≤ −0.8, gray edges) correlated with the expression levels of the lincRNAs. The network contains 68,625 edges among which 63,156 (92%) are positive correlations and 5469 (8%) negative ones. **(B)** A GO gene-enrichment analysis on the PC genes’ list revealed 16 significantly enriched GO terms (−log (p-value) on the x-axis), and among them there were membrane-related terms, as well as retrotransposons activity (orange dashed circles) and GPCR-associated pathways (purple dashed circles). The PC genes from the dash-circled GO categories were manually searched and clustered on the peripheral region from the network in (**A**), allowing one to see that both positive and negative correlations between lincRNA and PC genes are present on those processes.

A very often-adopted approach to hypothesize about lncRNAs’ function is the investigation of their co-expression patterns along with PC genes^12, 45, 48, 49^. We analyzed expression data for five different developmental stages of *S. mansoni* (15 RNA-Seq samples) in order to identify DE genes (previous topic), and we have selected a robust list of 181 SmLINCs that have strong evidence for being regulated genes. We therefore decided to perform an expression correlation analysis of these 181 lincRNAs against all DE PC genes (from the one way ANOVA-like heatmap on Fig. 3E) using read-counting values for each gene from the 15 RNA-Seq samples (biological triplicates for each life cycle stage). Among the correlated genes, we kept only gene pairs (lincRNA-PC gene) presenting *r* > 0.8 (classified as positively correlated) or *r* < −0.8 (negatively correlated). A co-expression network was built relying on that correlation information (Fig. 6A). It contains 181 lincRNAs (red nodes), 2359 PC genes (blue nodes), and 68,625 correlated pairs (edges), among which 92% (63,156/68,625) are for positive correlations (cyan edges) and 8% (5469/68,625) for negative ones (gray edges). As a negative control, a random network was generated with the same number of edges (68,625), 181 and 2359 randomly-picked lincRNAs and PC genes, respectively, in order to give support for the actual network significance. In this negative control dataset, we obtained 4788 edges that had either *r* ≥ 0.8 or *r* ≤ −0.8 out of 68,625 total edges (6.9%). Therefore, we could estimate a false discovery rate of 6.9% (4788/68,625), or a precision of ~ 93% for the correlations displayed on the actual network (see details on the Methods section).

A gene enrichment analysis for those 2359 PC genes was performed relying on the gene ontology (GO) terms assigned to them (see Methods). Sixteen GO categories comprise enriched genes with a significant hypergeometric test adjusted p-value < 0.01 (Fig. 6B). They are distributed into 10 cellular components (CC), 3 biological processes (BP) and 3 molecular functions (MF). Two terms from both BP and MF are highlighted: RNA-dependent DNA replication/RNA-directed DNA polymerase activity and G-protein coupled receptor (GPCR) signaling pathway/GPCR activity. The former represents genes involved in the transposition of retroelements, the major class of transposable elements (TEs) in *S. mansoni* genome, which is about 47% comprised by repeats^50^. TEs are also an important source for the birth of new lncRNAs within a genome^9^. The latter GO category is related to signal transduction via GPCR, which was already cited as a potential target in the context of drug discovery in *S. mansoni* ^51^. In addition, a recent study that built lncRNA-mRNA co-expression networks from venous congestion-subject human endothelial cells has identified GPCR as a potential pathway dynamically regulated by lincRNAs^48^.

### Filtering the network by topological proximity between SmLINC-PC gene pairs

Since it is known that lincRNAs may act by regulating their flanking-PC gene neighbors (as already mentioned herein), and that they may form triplex structures (dsDNA-RNA) anchoring on different genomic loci and recruiting chromatin remodelers such as PRC2 (refs 13, 14), we idealized a two-step filtering method in order to build a co-expression network where the lincRNA-PC gene expression correlations would be linked to the topological proximity of the pair on the chromatin. The first step was to filter each gene pair (lincRNA-PC gene) by a pre-defined correlation coefficient (*r*) threshold. Starting with the same input used for building the network on Fig. 6A, we have now decreased the cutoff to *r* ≥ 0.5 or *r* ≤ −0.5. The second step, which we will hereafter call topological filtering step (TFS), was aimed at keeping only the SmLINC-PC gene neighbors and/or the pairs presenting a triplex structure predicted by the triplexator algorithm^52^ (see Methods for details). After those two rounds of gene pairs filtering, we obtained a network containing 326 nodes (89 lincRNAs and 237 PC genes) that comprised 62.5% positively correlated pairs (204/326 edges) and 37.5% negatively correlated ones (122/326 edges) (Supplementary Fig. S3).

**Figure 7:**
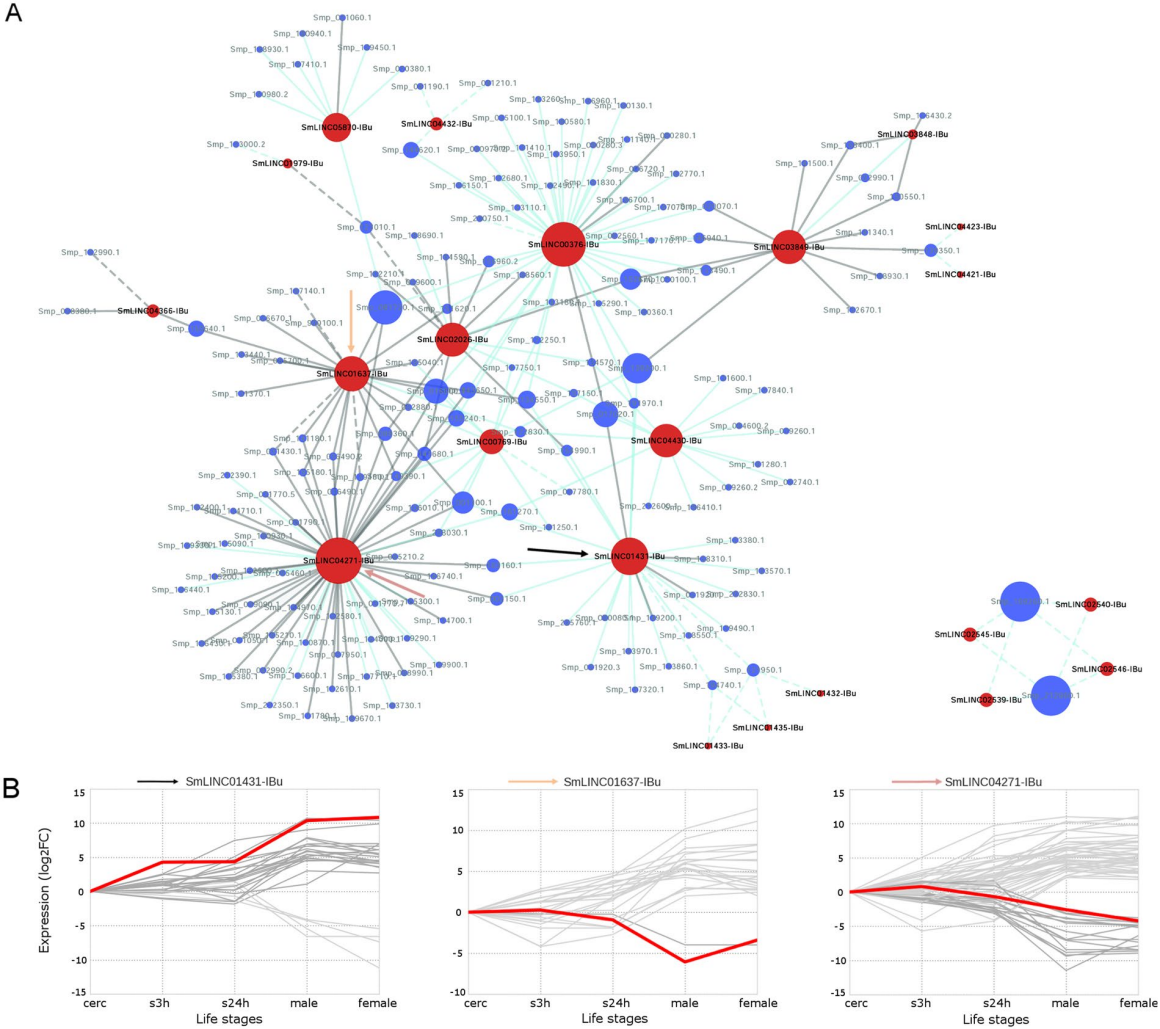
Topological filtering step (TFS) approach decreases the number of nodes and improves the visual inspection of lincRNAs’ subclusters. (**A**) Eighty nine out of the 181 robust lincRNAs from Fig. 4 passed at least one of the two TFS rules: (*i*) have a PC gene neighbor (Smp) as one of its correlated mates (putative *cis*-acting mechanism), and (*ii*) have an *in silico* computed probability to form a triplex structure (RNA-DNA) on its PC gene mate locus (putative *trans*-acting mechanism). The “Network Analyzer” function from cytoscape was used in order to obtain betweenness centrality score for each node, meaning the bigger the node, the higher propensity to be a hub. The 22 SmLINCs correlated with multiple PC genes are shown here. The entire network is shown on Supplementary Fig. S3 and it contains 326 nodes (89 SmLINCs and 237 Smps) with 62.5% positively correlated pairs (204/326, *r* ≥ 0.5, cyan edges) and 37.5% negatively correlated ones (122/326, *r* ≤ −0.5, gray edges). Dashed edges represent neighbor genes. (**B**) Gene expression patterns on three subnetworks that were manually selected by highlighting three SmLINC hubs, marked by arrows in (**A**) and named at the top of each panel in (**B**). Co-expression kinetics of the SmLINC (red line), positively correlated Smps (dark gray lines) and negatively correlated Smps (light gray lines) expressed across the five life cycle stages: cercariae (cerc), somula 3 h (s3h), somula 24 h (s24h), male and female.

We have also calculated the betweenness centrality (BC) score in order to identify important nodes within the network^53^. Roughly, BC measurement reflects the ratio of the number of shortest paths between two nodes (*x, z*) that pass through a node *y* by the number of total shortest paths between *x* and *z*, inferring, therefore, the propensity node *y* has to be a hub. The network layout that we show displays the node size proportional to the BC in each subnetwork (Fig. 7A and Supplementary Fig. S3). As examples, three subnetworks were selected and the expression profile across the five developmental stages of each target SmLINC and the correlated PC genes comprising each subnetwork have been displayed (Fig. 7B).

We performed a k-means clustering analysis on all the 326 genes present in the entire network, being able to detect that 70% of the genes (230/326) grouped into clusters that had an expression pattern similar to what was observed for the majority of the genes composing the SmLINC-hubs’ subclusters depicted on Fig. 7B, that is, an up-regulation profile from cercariae to adult forms (Supplementary Fig. S4). It appeared to be concurrent to what was recently reported about cercariae transcriptional poised state^42^.

### Another co-expression network building approach highlights PC gene hubs and their correlated lincRNAs

A second network building method, where we captured the most DE PC genes-correlated lincRNAs, was idealized. First we selected 307 PC genes (Smps) that were highly differentially expressed among the five life cycle stages on focus (log_2_FC ≥ 10 on at least one stage), and then we rescued all the other genes (Smps + SmLINCs) that presented a very stringent expression correlation (coefficient *r* ≥ 0.9 or *r* ≤ −0.9) with the 307 pre-chosen PC genes. A network with 2965 nodes (750 lincRNAs + 2215 PCs) and 52,913 edges (51,807 positive *r* and 1106 negative *r*) was generated by this approach (Supplementary Fig. S5 panel I). Due to their highest BC scores, three PC genes were detected as the most important nodes in the network: Smp_055340.2, Smp_038870.2 and Smp_036270.4. The first one (Smp_055340.2), with the highest BC value (~0.15, Supplementary Fig. S5 panel II), encodes the protein Lin-9, a nuclear tumor-suppressing agent in mammal cells^54^ that acts inhibiting DNA synthesis (G1/S transition). The second (Smp_038870.2) and third (Smp_036270.4) ones encode a NADH ubiquinone oxidoreductase and a splicing factor arginine/serine rich splicing factor 4, respectively. The expression patterns of SmLINCs that are either positively or negatively correlated with those three PC gene hubs along parasite differentiation are depicted on Supplementary Fig. S5 panel III.

Similarly to what was seen on the gene enrichment analysis for the 2359 Smps correlated with the 181 robust SmLINCs (Fig. 6A, B), the same assay performed on the different set of 2215 PC genes from this second network has also revealed “RNA-dependent DNA replication” and “DNA integration” as significantly enriched GO biological processes, as well as “Monovalent inorganic cation transmembrane transporter activity” as significantly enriched GO molecular functions (Supplementary Fig. S5 panel IV). Membrane and extracellular components’ terms were also significant in the gene enrichment.

Again, as a negative control we built a random network containing 2215 and 750 randomly-selected PC genes and lincRNAs, respectively, and the same number of edges present on the actual network. In this negative control, we retrieved 1550 out of 52,913 edges with *r* ≥ 0.9 or *r* ≤ −0.9, estimating a false discovery rate on the original network of 2.9%, or a precision of ~97%.

These results are indicative that SmLINC-Smp co-expression network construction may be considered as a reliable initial approach to hypothesize and direct further investigations on *S. mansoni* lincRNAs’ regulatory pathways.

## Discussion

Long non-coding RNAs are striking molecules acting on a variety of biological processes within the cell, mostly related to regulation of gene expression^2–4, 7, 8, 12, 14, 45^. Due to this broad landscape of content and functionality and despite the increasing efforts to unravel novel lncRNAs (mainly in higher eukaryotes), as well as their respective roles, the hitherto understanding of these molecules action on the diverse intracellular regulatory pathways still corresponds to a small tip of a huge iceberg. Regarding lncRNAs from more ancient important organisms, such as parasites, this tip is even smaller and few studies are devoted to unravel such genes.

The *S. mansoni* genome, which had its first version published in 2009 (ref. 51), and improved with the use of a NGS strategy in 2012 (ref. 21), has a size of about 380 Mb and nearly 12,000 PC genes (Smps) already mapped/annotated. These static data pose the even greater challenge of understanding the molecular dynamics responsible for the peculiar features of the parasite biology. Post-transcriptional control of gene expression events, such as modulation of mRNA alternative splicing and silencing by RNAi, as well as epigenetic events such as chromatin remodeling by histone modifications and the consequent epigenetic mechanisms to activate transcription, are present in schistosomes^41, 42, 55–57^. As in other higher eukaryotes, it is believed that a variety of lncRNAs may mediate such reactions in the parasites.

Regulatory RNAs appear to occur in all forms of life on the earth and it has been described that the protein-coding fraction of genomes decreases according to the complexity of organisms, ranging from 80–95% in prokaryotes to only a minor proportion in mammals (~1.22% in humans) (refs 58 and 59). On a brief screening on the latest version of *S. mansoni* genome and its current predicted proteome^21^, we detected a protein-coding DNA content of about 4.5%. Thus, it is apparent that *S. mansoni* has a considerable “free space” on the non-protein-coding portion of the genome which can be a source of hundreds to thousands lncRNAs that may act as regulatory and adaptive elements on the strenuous environmental changes this parasite experiences in order to complete its life cycle.

Due to the weak evolutionary constraint regarding lncRNAs’ sequence conservation^8, 9^, conventional local alignment search tools, commonly used for identifying homology evidence between PC genes from quite divergent organisms, are not the ideal ones for any kind of non-coding RNA sequence analyses^60^. With the advent of RNA-Seq, it is now possible to screen a whole transcriptome, on a high-throughput manner, in order to catch such transcripts. Both GENCODE^61^ and FANTOM^62^ consortia made use of the NGS strategy providing a great volume of input data to their computational pipelines aimed to assemble, distinguish/classify and annotate the maximum number of transcripts in humans and mice, respectively.

In the present study, we relied on tens of RNA-Seq samples (n = 88) from different developmental stages (Supplementary Table S6) and we built a transcriptomics/bioinformatics computational pipeline (Fig. 1) that successfully retrieved over 7,000*S. mansoni* multiexonic lncRNA transcripts (402 antisense to PC genes and 7029 lincRNAs) (Supplementary File S1 and Supplementary Table S1). It is important to note that our datasets do not represent the entire lncRNAs’ complement from the *S. mansoni* transcriptome, since we ruled out monoexonic transcripts in order to reduce the chance of artifacts^9^.

The conservative RNA-Seq assembly and filtering approach used here has shown 53% sensitivity when applied to the *P. falciparum* lincRNAs dataset^30^, as described in the Results. Therefore, it indicates that we are probably missing some *S. mansoni* lincRNA loci (most probably monoexonic ones), however we are certainly decreasing our false-positive rate by the stringent selection criteria established in our pipeline, which gives us a lower-boundary for the lincRNAs complement in our target *S. mansoni* organism.

The search for evidence of being active genes revealed only few hundreds lincRNAs (181) that have a combination of the four investigated traits: presence of H3K4me3 marks on their TSS-surrounding genomic regions, *Schistosoma spp*. phastCons conservation score assigned to at least one exon, differential expression across five developmental stages/forms (cercariae, somula 3 h, somula 24 h, male and female) and a significant either positive or negative expression correlation with their PC gene neighbors (Fig. 3). Sixteen out of those 181 lincRNAs had their expression individually quantified by us across the five parasite life stages/forms through RT-qPCR. It is noteworthy that, while some lincRNAs were found to be upregulated in adults (such as SmLINC02394-IBu and SmLINC06535-IBu, Fig. 5), most of the lincRNAs showed upregulation in schistosomula. This might indicate an importance of lincRNAs on regulating some processes involved in the rapid adaptation of schistosomula after transition from the free-living larvae to the early mammal parasitic stage, such as the worm body remodeling and defense against the host immune system.

We were guided by studies on mice and humans that make use of co-expression approaches on the attempt of hypothesizing lincRNAs’ function^12, 45, 49^. After clustering lincRNA-PC genes by either positive or negative expression correlation (roughly mimicking either activation/stabilization or inhibition/destabilization, respectively) across a set of different samples, co-expression networks were built and/or gene enrichment analyses were performed in order to acquire a holistic systems overview of potential lincRNAs’ roles. Two approaches were used for the networks construction that basically differ only on their initial dataset (list of genes) used for rescuing the additional positively or negatively correlated genes along parasite development. The first approach started with a list of 181 robust lincRNA candidates used for capturing their correlated Smp protein-coding genes (Figs 6 and 7), whereas the second one began with a list of 307 highly DE PC genes that were used for capturing their correlated genes in general (both SmLINCs and other Smps) (Supplementary Fig. S5). One interesting feature displayed by these two different network construction approaches was the presence of common GO categories of enriched PC genes: “RNA-dependent DNA replication”, “DNA integration”, “Monovalent inorganic cation transmembrane transporter activity” (Fig. 6B and Supplementary Fig. S5 panel IV), indicating that SmLINCs might be regulating such processes.

Both topological filtering step (TFS) and betweenness centrality (BC) measurement allowed us to highlight important SmLINCs (Fig. 7 and Supplementary Figs S3 and S5). For instance, SmLINC01637-IBu and SmLINC04271-IBu (orange and pink arrows on Fig. 7) appeared to be interesting candidates for functional studies. The former (SmLINC01637-IBu), while positively correlated to only one PC gene (ataxin 2 - Smp_122830), is negatively correlated to several mRNAs coding for membrane and/or secreted proteins, such as: fibrillin 2 (Smp_001100), solute carrier family 1 (Smp_016600), sodium dependent glucose transporter 1 (Smp_139150), transmembrane protein 26 (Smp_026670), transmembrane protein 231 (Smp_081720), fras1 related extracellular matrix protein (Smp_149390), protocadherin 9 (Smp_151620), surface membrane antigen (Smp_195180) and saposin B domain containing protein (Smp_016490.1 and 0.2), which has already been investigated as a vaccine candidate^63^. Whereas the latter (SmLINC04271-IBu) displays a mix of positively and negatively correlated mRNAs: more than half (8/15) on the first set (*r* ≥ 0.5) code for positively correlated hypothetical proteins (and noteworthy, the set includes a gene coding for the transcription factor forkhead box protein P1 - Smp_212350); and on the second set (*r* ≤ −0.5) there are 35% (15/43) and 39.5% (17/43) negatively correlated mRNAs coding for hypothetical proteins and membrane/secreted proteins, respectively, of which several are shared with SmLINC01637-IBu as negatively correlated (Smp_001100, Smp_016490.1, Smp_016490.2, Smp_016600, Smp_031430, Smp_081720, Smp_139150, Smp_141180, Smp_149390 and Smp_195180). Other interesting mRNAs are also listed on the *r* ≤ −0.5 set from SmLINC04271-IBu, such as SPARC protein (Smp_171780), PDZ domain-containing protein GIPC3 (Smp_170870), fasciclin domain-containing protein (Smp_141680), transmembrane protein 145 (Smp_125130), MEG-5 (Smp_152580) and a couple of transcription factors (HNF 4 - Smp_174700 and engrailed 2 C - Smp_145200).

This is the first time an approach for rescuing lncRNAs plus another for proposing their possible functionality through co-expression networks’ construction are reported in a single study on a parasite’s genome/transcriptome landscape. Adding to what was already seen for *P. falciparum* regarding lncRNAs^30, 64^, the data presented herein reinforce that those transcripts are expressed and may play a role on the biology of neglected tropical disease-causing agents. Thus, parasites’ lncRNAs and their pathways of action should start being considered as possible new therapeutic targets on future investigations.

## Conclusions

The established computational pipeline appears to be a robust tool for the identification of thousands of multiexonic *S. mansoni* lncRNAs and might be applicable to any other organism. Hundreds of the putative lincRNAs display evolutionary conservation within the *Schistosoma* genus, transcriptional activation epigenetic mark (H3K4me3) at their TSSs, differential expression across five developmental stages and expression correlation with their protein-coding gene neighbors. In addition, RT-qPCR assays for 16 SmLINCs have confirmed that they undergo differential expression along the parasite development.

The construction of co-expression networks allows a holistic systems overview that helps us to decipher the role of these intriguing molecules on *S. mansoni* biology. We believe that the networks built and disclosed herein have now paved the way, as an important initial source, for investigations on *S. mansoni* regulatory pathways involving lincRNAs and their correlated PC genes. Functional assays are necessary to characterize individual SmLINCs as regulatory elements of their PC gene counterparts within the same co-expression subcluster.

## Methods

Full methods are available on-line in the Supplementary Materials section.

### Ethics statement

All protocols involving animals were conducted in accordance with the Ethical Principles in Animal Research adopted by the Brazilian College of Animal Experimentation (COBEA), and the protocol/experiments have been approved by the Ethics Committee for Animal Experimentation of Instituto Butantan (CEUAIB Protocol number 1777050816).

### Genomic and transcriptomic data

The *Schistosoma* genomes analyzed herein were downloaded from the Wellcome Trust Sanger Institute ftp site for *S. mansoni* (http://ftp.sanger.ac.uk/pub/pathogens/Schistosoma/mansoni/Latest_assembly_annotation_others/Schistosoma_mansoni_v5.2.fa) and from wormbase ftp for both *S. haematobium* (http://ftp.ebi.ac.uk/pub/databases/wormbase/parasite/releases/WBPS8/species/schistosoma_haematobium/PRJNA78265/schistosoma_haematobium.PRJNA78265.WBPS8.genomic.fa.gz) and *S. japonicum* (http://ftp.ebi.ac.uk/pub/databases/wormbase/parasite/releases/WBPS8/species/schistosoma_japonicum/PRJEA34885/schistosoma_japonicum.PRJEA34885.WBPS8.genomic.fa.gz). The most recent genomic annotation of nearly 12,000 PC genes (Smps) was used^21^.

We have used 88 *S. mansoni* RNA-Seq libraries as input for our computational pipeline (more details on the next topic): 52 from public studies already deposited in the Sequence Read Archive (SRA) NCBI database, which encompass several developmental stages (Supplementary Table S6), and 36 in-house separate male (Accession numbers SAMN06221530-SAMN06221541 and SAMN06221554-SAMN06221559) and female libraries (Accession numbers SAMN06221542-SAMN06221553 and SAMN06221560-SAMN06221565).

### Assembly of RNA-Seq reads

Trinity *de novo* assembly^32^ was applied to three different RNA-Seq datasets: (1) trin_strA dataset, comprised of ~300 million raw paired-end reads (SRA accession SAMN06221530-SAMN06221553) obtained in house from 24 samples of adult worm couples exposed *in vitro* to TH65 (compound 13I) (ref. 65) or to vehicle (controls); (2) trin_strB dataset, comprised of ~300 million raw paired-end reads (SRA accession SAMN06221554-SAMN06221565), again obtained in house from 12 samples of adult worm couples exposed *in vitro* to GSK343 (ref. 66) or to vehicle (controls); (3) trin_sra dataset, which encompasses 52 RNA-Seq libraries (Supplementary Table S6) (~2 billion raw reads) from *S. mansoni* at several different life cycle stages (both untreated samples or samples treated with different compounds), which were downloaded from the SRA-NCBI public repository. A second assembly approach using the Tuxedo tools (Tophat2 (ref. 33), Cufflinks and Cuffmerge^34^) was applied to a subset of the above RNA-Seq libraries that included 44 out of the 52 SRA RNA-Seq libraries comprised exclusively of control untreated samples, plus 12 in house-derived libraries from both strA and strB comprised exclusively of control samples (total of ~2.2 billion raw reads). Each of those 56 samples (44 + 12) was used as an independent input file for both tophat2 and cufflinks, and then all 56 independently assembled transcript sets were merged onto a single non-redundant gtf file through cuffmerge execution. See further details in the Supplementary Materials section.

### Retrieving long non-coding RNAs from the assembled transcripts’ dataset

After having all the assembled transcripts mapped to the reference genome (bed12 and gtf formats for *de novo* and genome-guided assemblies, respectively), a series of filtering steps was applied as described below, aimed at both removing unwanted protein-coding transcripts and rescuing putative lncRNAs from the entire transcriptome datasets (red rectangles section from the pipeline depicted on Fig. 1). The tools that were used and their respective parameters were all placed in one single PERL script and adapted for automation; the script can be downloaded from https://github.com/eltonjrv/Smansoni.lncRNAs. See further details in the Supplementary Materials section.

### Searching for evidence supporting *S. mansoni* lincRNAs as functional genes

#### H3K4me3 marks

In order to search for the presence of histone H3 lysine 4 trimethylation (H3K4me3) on the transcription start site (TSS) of *S. mansoni* genes as an epigenetic mark for transcriptional activation, we relied on four ChIP-Seq assays publicly available at the SRA-NCBI database: SRR1107840 and SRR2530135 were obtained from adult worms, whereas SRR2120359 and SRR2120360 from schistosomula parasites. Those data were all generated by the same group and are part of three different publications^42, 67, 68^. See further details in the Supplementary Materials section.

#### Evolutionary conservation by whole genomes’ comparison

In order to mask both repeats and low complexity regions present within *Schistosoma spp*. genomes, we ran RepeatMasker (http://repeatmasker.org) (-e crossmatch -pa 20 -q -xsmall -gff -norna -lib RepBasePerpignanSma52.fasta) on each of the three whole genomes compared herein. Pairwise alignments of *S. mansoni* repeat-masked genome against *S. haematobium* and *S. japonicum* ones were performed using lastz algorithm (https://www.bx.psu.edu/~rsharris/lastz/), an improved version of blastz^69^. See further details in the Supplementary Materials section.

#### Differential Expression and correlation analyses across five developmental stages

Fifteen RNA-Seq libraries from five different developmental stages of *S. mansoni* (biological triplicate each) were selected for investigation of gene expression profile of both PC and lncRNA genes: cercariae (ERR022872, ERR022877 and ERR022878), somula 3 h (ERR022874, ERR022876 and ERR022879), somula 24 h (ERR022880, ERR022881 and ERR022882), male (SAMN06221530, SAMN06221531 and SAMN06221532) and female (SAMN06221542, SAMN06221543 and SAMN06221544) adults. See further details in the Supplementary Materials section.

### Co-expression network construction and analyses

Based on the general gene expression correlation analysis among *S. mansoni* five developmental stages (described on the topic just above), we were able to retrieve lincRNA-PC gene pairs that are either positively or negatively correlated by selecting arbitrary *r* thresholds (see *r* cutoffs established by us further in this topic). We used Unix/Shell tools on the output of the ad-hoc correlation’s R script in order to prepare the simple interaction formats (.sif) to feed Cytoscape software^70^ for both network visualization and further analyses within the network. For each correlated genes’ pair we assigned a “pos” or “neg” edge name regarding whether the correlation between the genes is positive or negative, respectively. See further details in the Supplementary Materials section.

### Statistical analyses and charts plotting

Statistical analyses and charts plotting were performed within the R environment (version 3.3.2) with limma, edgeR, gplots and ggplot2 libraries loaded. Gene expression line charts were generated using matplotlib in Python. The intersection diagram in the UpSet format^71^ was plotted using Intervene (https://asntech.shinyapps.io/intervene/) (doi:10.1101/109728).

### Parasite materials

All parasite material was from a BH isolate of *S. mansoni* maintained by passage through golden hamster (*Mesocricetus auratus*) and *Biomphalaria glabrata* snails. See further details in the Supplementary Materials section.

### RNA extraction, quantification and quality assessment

Total RNA from cercariae (C) and schistosomula (3S and 24S) was extracted using a protocol based on Roquis *et al*., 2015 (ref. 42). Briefly, 25,000 cercariae or schistosomula were ground with glass beads in liquid nitrogen for 5 minutes. Then the Qiagen RNeasy Micro Kit (Cat number 74004) was used for RNA extraction and purification according to the manufacturer’s instructions, except for the DNase I treatment: the amount of DNase I was doubled and the time of treatment was increased to 45 minutes. Male or female worms were first disrupted in Qiagen RLT buffer using glass potters and pestles. RNA from males and females was then extracted and purified using the Qiagen RNeasy Mini Kit (Cat number 74104), according to the manufacturer’s instructions, except for the DNase I treatment, which was the same used for cercariae and schistosomula RNA extraction. All the RNA samples were quantified using the Qubit RNA HS Assay Kit (Q32852, Thermo Fisher Scientific) and the integrity of RNAs was verified using the Agilent RNA 6000 Pico Kit (5067-1513 Agilent Technologies) in a 2100 Bioanalyzer Instrument (Agilent Technologies). Four biological replicates were assessed for each life cycle stage.

### RT-qPCR assays

The reverse transcription (RT) reaction was performed with 100 ng of each total RNA sample using the SuperScript IV First-Strand Synthesis System (18091050, Life Technologies) and random hexamer primers in a 20 μL final volume. The obtained complementary DNAs (cDNAs) were diluted 10 times in water and quantitative PCR was performed using 2.5 μL of each diluted cDNA in a total volume of 10 μL containing 1X LightCycler 480 SYBR Green I Master Mix (04707516001, Roche Diagnostics) and 800 nM of each primer in a LightCycler 480 System (Roche Diagnostics). Primers for selected transcripts (Supplementary Table S5) were designed using the Primer 3 tool (http://biotools.umassmed.edu/bioapps/primer3_www.cgi), and each real-time qPCR was run in three technical replicates. The results were analyzed by comparative Ct method^72^. The reference gene Smp_092920 was chosen from twelve genes that showed no differential expression in the RNA-Seq data along the five stages. Data from the RT-qPCR expression values of the twelve genes across the five stages were analyzed with RefFinder^73^ (http://150.216.56.64/referencegene.php) using three tools (BestKeeper, NormFinder and GeNorm) in order to choose the most stable gene for qPCR. Real-time data were normalized according to the expression level of the Smp_092920 reference gene, and p-values were determined by one-way analysis of variance (ANOVA) and Tukey post-hoc tests.

### Data Availability

The RNA-seq datasets generated during the current study are available in the Sequence Read Archive (SRA) NCBI repository under Accession numbers SAMN06221530-SAMN06221541, SAMN06221554-SAMN06221559, SAMN06221542-SAMN06221553 and SAMN06221560-SAMN06221565. A GTF file with the *S. mansoni* lincRNAs identified with our pipeline can be downloaded from http://verjolab.usp.br/tracks/schMan/schMan1/. All other public RNA-seq data analyzed during the current study are from the SRA repository and their Accession numbers are listed in Supplementary Table S6.

## Electronic supplementary material

Supplementary Methods

Table S1

Table S2

Table S3

Table S4

Table S5

Table S6

File S1

File S2
