# Supplementary material for: The Schistosoma mansoni genome encodes thousands of long non-coding RNAs predicted to be functional at different parasite life-cycle stages
Source: Sci Rep. 2017 Sep 5;7:10508. doi: 10.1038/s41598-017-10853-6 (PMC5585378; doi:10.1038/s41598-017-10853-6)
Supplement: Supplementary file 1 — Supplementary Methods [file 41598_2017_10853_MOESM1_ESM.pdf]

Supplementary Methods and Figures for

The *Schistosoma mansoni* genome encodes thousands of long non-coding RNAs predicted to be functional at different parasite life-cycle stages

Elton J. R. Vasconcelos[1,2,#], Lucas F. daSilva[1,2], David S. Pires[1], Guilherme M. Lavezzo[1,2], Adriana S. A. Pereira[1,2], Murilo S. Amaral[1] and Sergio Verjovski-Almeida[1,2*]

[1] Laboratório de Expressão Gênica em Eucariotos, Instituto Butantan, 05503-900 São Paulo, SP, Brazil

[2] Departamento de Bioquímica, Instituto de Química, Universidade de São Paulo, 05508-900 São Paulo, SP, Brazil

[#] Current address: College of Veterinary Medicine, Western University of Health Sciences, Pomona, CA, USA

* Corresponding author

E-mail: verjo@iq.usp.br (SVA)

Index

**Supplementary Methods**	page 2
**Fig. S1**	page 9
**Fig. S2**	page 10
**Fig. S3**	page 11
**Fig. S4**	page 12
**Fig. S5– panel I**	page 13
**Fig. S5 – panels II, III, IV**	page 14
**Fig. S6**	page 15
**Supplementary References**	page 16

Supplementary Methods

Assembly of RNA-Seq reads

Trinity *de novo* assembly[1] was applied to three different RNA-Seq datasets: (1) trin_strA dataset, comprised of ~ 300 million raw paired-end reads (SRA accession SAMN06221530-SAMN06221553) obtained in house from 24 samples of adult worm couples exposed *in vitro* to TH65 (ref. 2) or to vehicle (controls); (2) trin_strB dataset, comprised of ~ 300 million raw paired-end reads (SRA accession SAMN06221554-SAMN06221565), again obtained in house from 12 samples of adult worm couples exposed *in vitro* to GSK343 (ref. 3) or to vehicle (controls); (3) trin_sra dataset, which encompasses 52 RNA-Seq libraries (Supplementary Table S5) (~ 2 billion raw reads) from *S. mansoni* at several different life cycle stages (both untreated samples or samples treated with different compounds), which were downloaded from the SRA-NCBI public repository. A second assembly approach using the Tuxedo tools (Tophat2 (ref. 4), Cufflinks and Cuffmerge[5]) was applied to a subset of the above RNA-Seq libraries that included 44 out of the 52 SRA RNA-Seq libraries comprised exclusively of control untreated samples, plus 12 in house-derived libraries from both strA and strB comprised exclusively of control samples (total of ~ 2.2 billion raw reads). Each of those 56 samples (44 + 12) was used as an independent input file for both tophat2 and cufflinks, and then all 56 independently assembled transcript sets were merged onto a single non-redundant gtf file through cuffmerge execution.

After checking the libraries' quality with FastQC (http://www.bioinformatics.babraham.ac.uk/projects/fastqc/) and trimming adapters and low quality bases (phred score < 20) with Trimmomatic[6], the following parameters were used for both *de novo* and genome-guided assemblies:

- *de novo* (Trinity transcript assembler):

Trinity v2.2.0 parameters for in house libraries: --CPU 32 --max_memory 216G --SS_lib_type RF --seqType fq --min_contig_length 200;

Trinity v2.2.0 parameters for SRA libraries: --CPU 32 --max_memory 216G --seqType fq --single --run_as_paired --min_contig_length 200;

Blat[7] parameters (trinity contigs againstr *S. mansoni* genome): -stepSize=5 -t=dna -q=rna;

pslToBed script from kentUtils (https://github.com/ENCODE-DCC/kentUtils/tree/master/src/hg/pslToBed) was run for converting psl Blat output to bed12 format.

- Genome-guided (Tuxedo tools):

Tophat2 v2.1.1 parameters: --min-intron-length 10 --max-intron-length 30000 --library-type [fr-firststrand or fr-unstranded] --b2-very-sensitive --GTF SMPs-withUTRs-5.2-woSm-wParent-NEW.gff --num-threads 26 Schistosoma_mansoni_v5.2;
Cufflinks v2.2.1 parameters: -p 20 -g SMPs-withUTRs-5.2-woSm-wParent-NEW.gff --multi-read-correct --frag-bias-correct Schistosoma_mansoni_v5.2.fa --min-intron-length 10;
Cuffmerge (an executable of the Cufflinks package) parameters: -p 20 -o cuffmergeOUT -g SMPs-withUTRs-5.2-woSm-wParent-NEW.gff -s Schistosoma_mansoni_v5.2-woSm.fa transcripts-GTF.fofn.

Retrieving long non-coding RNAs from the assembled transcripts' dataset

After having all the assembled transcripts mapped to the reference genome (bed12 and gtf formats for *de novo* and genome-guided assemblies, respectively), a series of filtering steps was applied as described below, aimed at both removing unwanted protein-coding transcripts and rescuing putative lncRNAs from the entire transcriptome datasets (red rectangles section from the pipeline depicted on Fig. 1). The following tools and their respective parameters were all placed in one single PERL script and adapted for automation; the script can be provided upon request.

Bedtools v2.25.0 (ref. 8) parameters: intersect –v (for rescuing lincRNAs) or –S –loj –f 0.33 (for rescuing anti-sense lncRNAs) –b SMPs-withUTRs-5.2.bed;
RepeatMasker version open-3.1.5 (http://repeatmasker.org) parameters: -s -lib RepBasePerpignanSma52-plusDNAtranspNCBI.fasta -x -gff -gc 35 -pa 12;
Ribopicker v0.4.3 (ref. 9) parameters: -i 70 -c 50 -dbs rrnadb;
Shell (bash) tools and an ad-hoc PERL script were executed in order to retrieve transcripts that have at least one intron greater than 30 nt and canonical splicing sites on all junctions;
Getorf (an executable from the EMBOSS v6.6.0.0 package) (ref. 10) parameters: -noreverse -minsize 75 -find 0;
CPC v0.9-r2 (ref. 11) and TransDecoder v2.0.1 (http://transdecoder.github.io/) were run using default parameters;
Interproscan v5.19 (ref. 12) paramaters: -appl Panther,Pfam -t n -goterms –iprlookup.
At the end of this mining batch pipeline, we obtained a general dataset of *S. mansoni* putative lncRNAs.

Searching for evidence supporting *S. mansoni* lincRNAs as functional genes

H3K4me3 marks

In order to search for the presence of histone H3 lysine 4 trimethylation (H3K4me3) on the transcription start site (TSS) of *S. mansoni* genes as an epigenetic mark for transcriptional activation, we relied on four ChIP-Seq assays publicly available at the SRA-NCBI database: SRR1107840 and SRR2530135 were obtained from adult worms, whereas SRR2120359 and SRR2120360 on schistosomula forms. Those data were all generated by the same group and are part of three different publications[13-15].

We adopted the same computational pipeline described by Anderson et al., 2015 for mapping those marks on *S. mansoni* genome[16] and statistically assigning the significant peaks[17]. Bedtools[8] (window -c -l 1000 -r 1500 -sw) was used for capturing genes (both PC genes and lincRNAs) that have at least one significant peak on their flanking regions (1 kb upstream and 1.5 kb downstream the TSS).

Evolutionary conservation by whole genomes' comparison

In order to mask both repeats and low complexity regions present within *Schistosoma spp.* genomes, we ran RepeatMasker (http://repeatmasker.org) (-e crossmatch -pa 20 -q -xsmall -gff -norna -lib RepBasePerpignanSma52.fasta) on each of the three whole genomes compared herein. Pairwise alignments of *S. mansoni* repeat-masked genome against *S. haematobium* and *S. japonicum* ones were performed using lastz algorithm (https://www.bx.psu.edu/~rsharris/lastz/), an improved version of blastz[18]. The following commands were used to obtain pairwise genome alignments (Sman vs Shae and Sman vs Sjap) on a maf format.

```
$ blastzWrapper Smansoni Shaematobium Y=9000 H=0   >Smansoni.Shaematobium.lav
$ lav2maf Smansoni.Shaematobium.lav Smansoni Shaematobium
>Smansoni.Shaematobium.maf
$ maf_sort Smansoni.Shaematobium.maf Smansoni > Smansoni.Shaematobium.orig.maf
$ blastzWrapper Smansoni Sjaponicum Y=9000 H=0   >Smansoni.Sjaponicum.lav
$ lav2maf Smansoni.Sjaponicum.lav Smansoni Sjaponicum  >Smansoni.Sjaponicum.maf
$ maf_sort Smansoni.Sjaponicum.maf Smansoni > Smansoni.Sjaponicum.orig.maf
```

The multiple alignment arrangement was generated by running TBA[19] (tba E=Smansoni "((Smansoni Shaematobium) Sjaponicum)" *.*.maf schistos_pub-tba.maf) in order to get a single maf file containing all the aligned genomic stretches from the three species that are used as input for phastCons algorithm[20] (phastCons --target-coverage 0.25 --expected-length 12 --estimate-trees treeSchistos_pub --msa-format MAF Chr_1-RandomSample.maf init.mod --no-post-probs). Noteworthy that, before running phastCons, a phylogenetic tree model had to be constructed with phyloFit[21] (phyloFit --tree "((Smansoni,Shaematobium),Sjaponicum)" --msa-format MAF --out-root init schistos_pub-tba-cleaned.maf).

Differential Expression and correlation analyses across five developmental stages

Fifteen RNA-Seq libraries from five different developmental stages of S. mansoni (biological triplicate each) were selected for investigation of gene expression profile of both PC and lncRNA genes: cercariae (ERR022872, ERR022877 and ERR022878), somula 3h (ERR022874, ERR022876 and ERR022879), somula 24h (ERR022880, ERR022881 and ERR022882), male (SAMN06221530, SAMN06221531 and SAMN06221532) and female (SAMN06221542, SAMN06221543 and SAMN06221544) adults.

Read counts and “transcripts per million” normalized counts (TPM) were assessed by running kallisto[22] (-t 8 –b 50) against an indexed reference transcriptome file that comprised all 11,876 already annotated PC genes (Smp) and the 7029 putative lincRNAs identified herein. In order to avoid bias due to very low expression levels some genes may have (mainly lincRNAs), we decided to select only the genes that showed TPM ≥ 1 on all the three replicates of at least one life cycle stage. We retrieved 10,482 Smps and 2091 lincRNAs that served as input for the one-way ANOVA-like method from the edgeR suite[23] (following the default parameters described on the edgeR user guide, last revised on Jun/30/2016), which were used for detecting differentially expressed genes across the five parasite forms (adjusted p-value < 0.01). Cercariae were set as the intercept group. A multi-dimensional scaling plot was generated by edgeR before performing the one-way ANOVA-like in order to check the samples convergence/divergence (Supplementary Fig. S6).

Besides the differential expression analysis, we have also performed Pearson Correlation Coefficient (*r*) assessment on the genes having TPM ≥ 1 (10,482 Smps and 2091 lincRNAs = 12,573 genes) using the TPM values for each gene from the 15 libraries in the following order: cercariae, somula 3h, somula 24h, male and female (biological triplicate each). An R script was

written to automatically assign *r* to all possible gene pairs' combination within the list. The output of that script was used as the main source for further analyses involving expression correlation, such as the screening for correlation between lincRNA-PC gene neighbors and the co-expression network construction (described below).

Co-expression network construction and analyses

Based on the general gene expression correlation analysis among *S. mansoni* five developmental stages (described on the topic just above), we were able to retrieve lincRNA-PC gene pairs that are either positively or negatively correlated by selecting arbitrary *r* thresholds (see *r* cutoffs established by us further in this topic). We used Unix/Shell tools on the output of the ad-hoc correlation's R script in order to prepare the simple interaction formats (.sif) to feed Cytoscape software[24] for both network visualization and further analyses within the network. For each correlated genes' pair we assigned a "pos" or "neg" edge name regarding whether the correlation between the genes is positive or negative, respectively.

We idealized two approaches for the co-expression network construction. *(i)* The first one was based on the selection of the 181 lincRNAs (red nodes) that shared all the four traits for being functional (H3K4me3 mark at their TSSs, phastCons evolutionary conservation score, differentially expressed across the five stages and with expression correlated with that of its PC gene neighbor) and then rescuing their positively (cyan blue edges) or negatively (gray edges) correlated PC gene counterparts (blue nodes). At first we chose an *r* cutoff of $r \geq 0.8$ or $r \leq -0.8$, which retrieved 2359 PC genes (see Fig. 6 and Results section). Subsequently, we established a two-steps filtering method in order to rescue lincRNA-PC gene pairs that might be topologically close to each other, which we called topological filtering step (TFS). For the TFS we decreased the *r* threshold to $r \geq 0.5$ or $r \leq -0.5$ (first step) and then filtered the pairs by obeying one of the following two rules: the lincRNA and the PC genes are either neighbors (considering the first two PC genes upstream and two downstream of each lincRNA) or the lincRNA may form a putative triplex structure on their co-expressed PC loci within the genome (the entire PC locus plus 1 kb upstream of its TSS). The triplex structure predictions were obtained by running triplexator v1.3.2 (ref. 25) (-mf –dl). Applying that topological filtering strategy we obtained a much smaller network with 89 out of the 181 lincRNAs and 237 Smps (see Fig. 7 and Results section). *(ii)* The second approach for a co-expression network construction was based on the selection of 319 highly differentially expressed PC genes (yellow nodes), which showed a

$log_2FC \geq 10$ on at least one out of the five developmental stages, and then rescuing their positively ($r \geq 0.9$, cyan blue edges) or negatively ($r \leq -0.9$, gray edges) correlated lincRNAs (red nodes) and/or other PC genes (blue nodes). Three hundred and seven out of the 319 selected PC genes were correlated with at least one lincRNA, so they were the genes used to build the network. This network contained 2215 PC genes and 750 lincRNAs (see Supplementary Fig. S5 and Results section).

The "Network Analyzer" tool from Cytoscape was used in order to calculate the betweenness centrality (BC) values[26] for each node on the networks built herein (see Results for more details).

To further test the significance of the networks built on both approaches described above, we adopted the same method used by Necsulea et al., 2014 (ref. 27). We randomly-picked expressed lincRNAs and PC genes (TPM ≥ 1 on at least one out of the five developmental stages on focus) in order to build a random network presenting the same architecture of the actual one, that is, same number of lincRNAs, PC genes and edges (keeping the same number of edges that connect each lincRNA to their PC gene counterparts on the actual network). This strategy showed us a false discovery rate less than 7% for both network construction approaches, indicating that our construction methods have a precision (positive predictive value) greater than 93%.

The hypergeometric test for Gene Ontology (GO) gene enrichment analyses on the PC genes present on the co-expression networks was performed by BinGO[28], a Cytoscape plugin, using an adjusted p-value cutoff of 0.01.

Parasite materials

All parasite material was from a BH isolate of *S. mansoni* maintained by passage through golden hamster (*Mesocricetus auratus*) and *Biomphalaria glabrata* snails. Cercariae were collected from snails infected with 10 miracidia each. Thirty-five days after infection, the snails were placed in the dark in water and then illuminated for two hours to induce shedding. The emerging cercariae were cooled on ice for 30 minutes to prevent swimming and collected by centrifugation, washed with water once and then stored in RNAlater (Ambion) until RNA extraction. Schistosomula were obtained by mechanical transformation of cercariae and separation of their bodies as previously described[29] , with some modifications. Briefly, cercariae

were collected as described above and then suspended in 15 ml of M169 medium (Vitrocell, cat number 00464) containing penicillin/streptomycin, amphotericin (Vitrocell, cat number 00148). Mechanical transformation was performed by passing the cercariae ten times through a 23G needle. In order to separate schistosomula from the tails, the tail-rich supernatant was decanted and the sedimented bodies resuspended in a further 7 ml of M169 medium. The procedure was repeated until less than 1% of tails remained. The newly transformed schistosomula were maintained for 3h or 24h in M169 medium (Vitrocell, cat number 00464) supplemented with penicillin/streptomycin, amphotericin, gentamicin (Vitrocell, cat number 00148), 2% fetal bovine serum, 1 μM serotonin, 0.5 μM hypoxanthine, 1 μM hydrocortisone and 0.2 μM triiodothyronine at 37°C and 5% CO2. Schistosomula cultivated for 3h (3S) or for 24h (24S) were washed 3 times with PBS and stored in RNAlater (Ambion) until RNA extraction.

Adult *S. mansoni* worms were recovered by perfusion of golden hamsters that had been infected with 200-300 cercariae, 7 weeks previously. Approximately 200 *S. mansoni* (BH strain) adult worm pairs were freshly obtained through the periportal perfusion of hamster, as previously described [16]. After perfusion, the adult worm pairs were kept for 3 hours at 37°C and 5% CO2 in Advanced RPMI Medium 1640 (Gibco, #12633–012) supplemented with 10% heat-inactivated calf serum (freshly added), 12 mm HEPES (4-(2-hydroxyethyl) piperazine-1-ethanesulfonic acid) pH 7.4, and 1% penicillin/streptomycin, amphotericin (Vitrocell, cat number 00148). After 3h of incubation, the adult worm pairs were washed 3 times with PBS and stored in RNAlater (Ambion) for further RNA extraction. Right before RNA extraction, adult worm pairs were manually separated in RNAlater (Ambion) using tweezers in order to obtain male- and female-only RNA samples.

Supplementary Figures

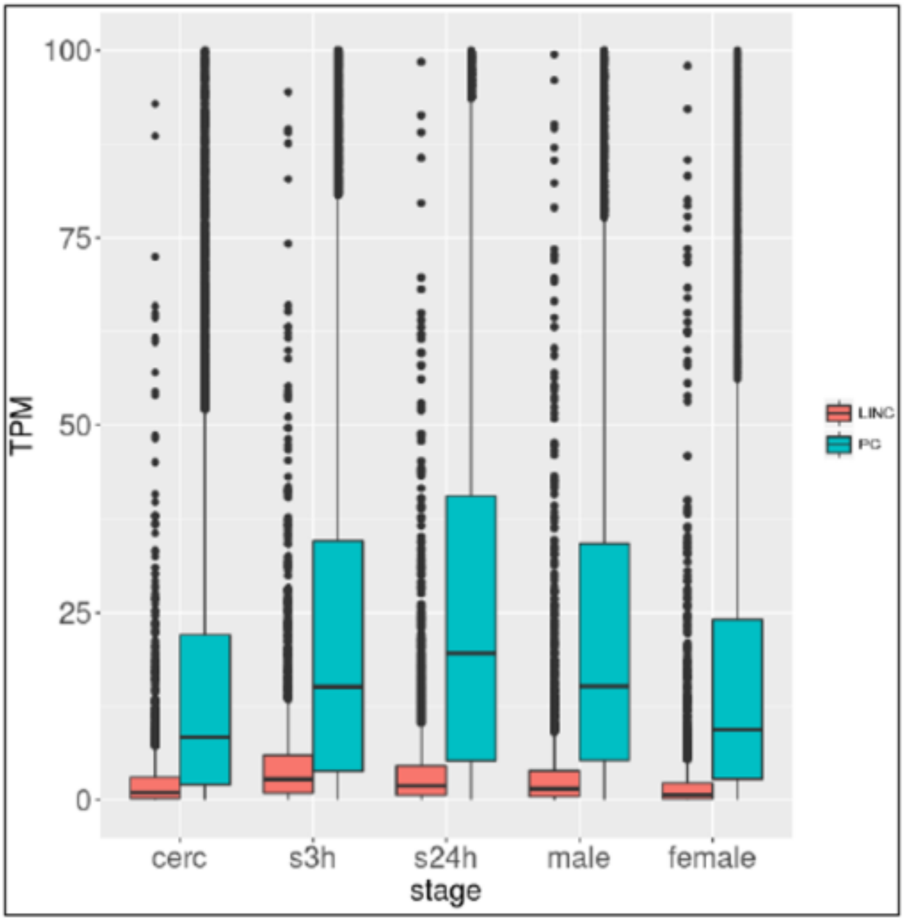

T-test p-values: 1.1e-09 1.2e-20 7.4e-17 1.6e-41 5.1e-12

Figure S1. SmLINCs have significantly lower expression when compared to protein-coding Smp genes on five different developmental stages. The **SmLINC** genes lists (red) and the **protein-coding Smp genes lists (cyan)** were filtered by TPM ≥ 1. Y axis: normalized expression levels (transcripts per million, TPM). X axis: cercariae (cerc), somula 3h (s3h), somula 24h (s24h), male and female forms of the parasite. t-test p-values < 1e-08.

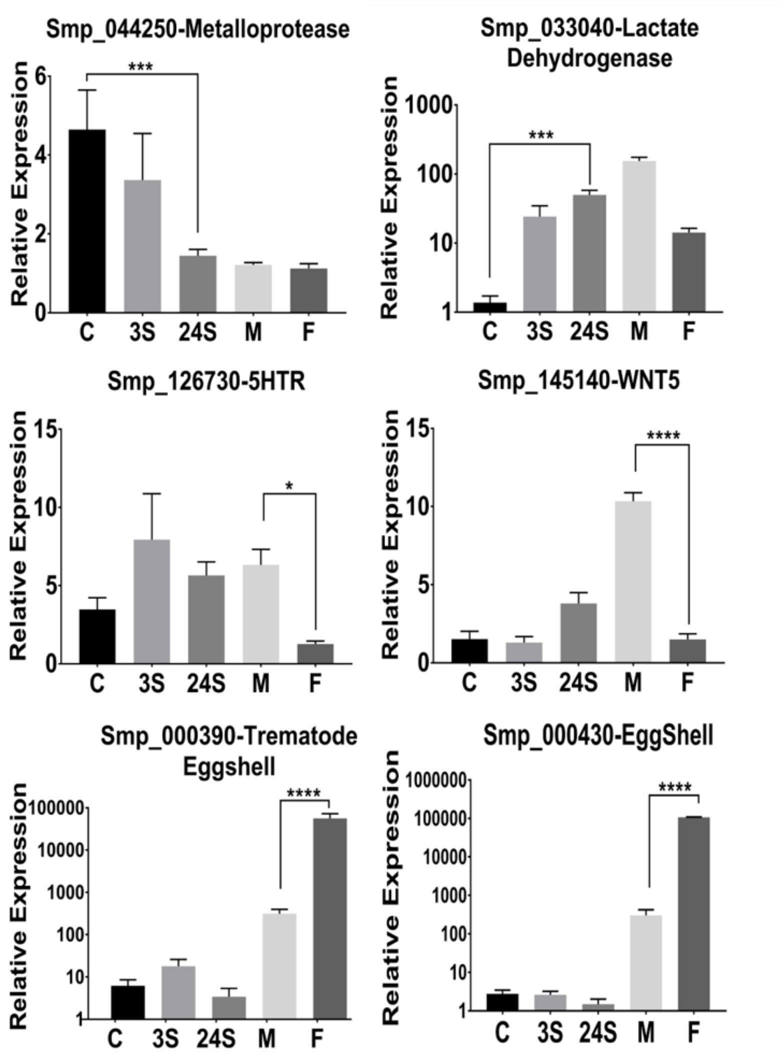

Figure S2. Confirmation by RT-qPCR of the differential expression of six protein-coding genes selected as positive controls. Smp_044250 (Metalloprotease), shown as up-regulated in cercariae compared with somula[30]; Smp_033040 (Lactate dehydrogenase), up-regulated in somula compared with cercariae[30]; Smp_126730-5HTR and Smp_145140-WNT5, assessed as up-regulated in adult male compared with female[16];, Smp_000390-Trematode Eggshell and Smp_000430-EggShell, up-regulated in adult female compared to male[16]. RT-qPCR assays were carried out on five parasite stages/forms, namely cercariae (C), schistosomula after 3 hours of mechanical transformation (3S), schistosomula after 24 hours of mechanical transformation (24S), adult male and female. The relative expression measurements, as well as the number of replicates and statistical analyses were the same as described in Fig 5.

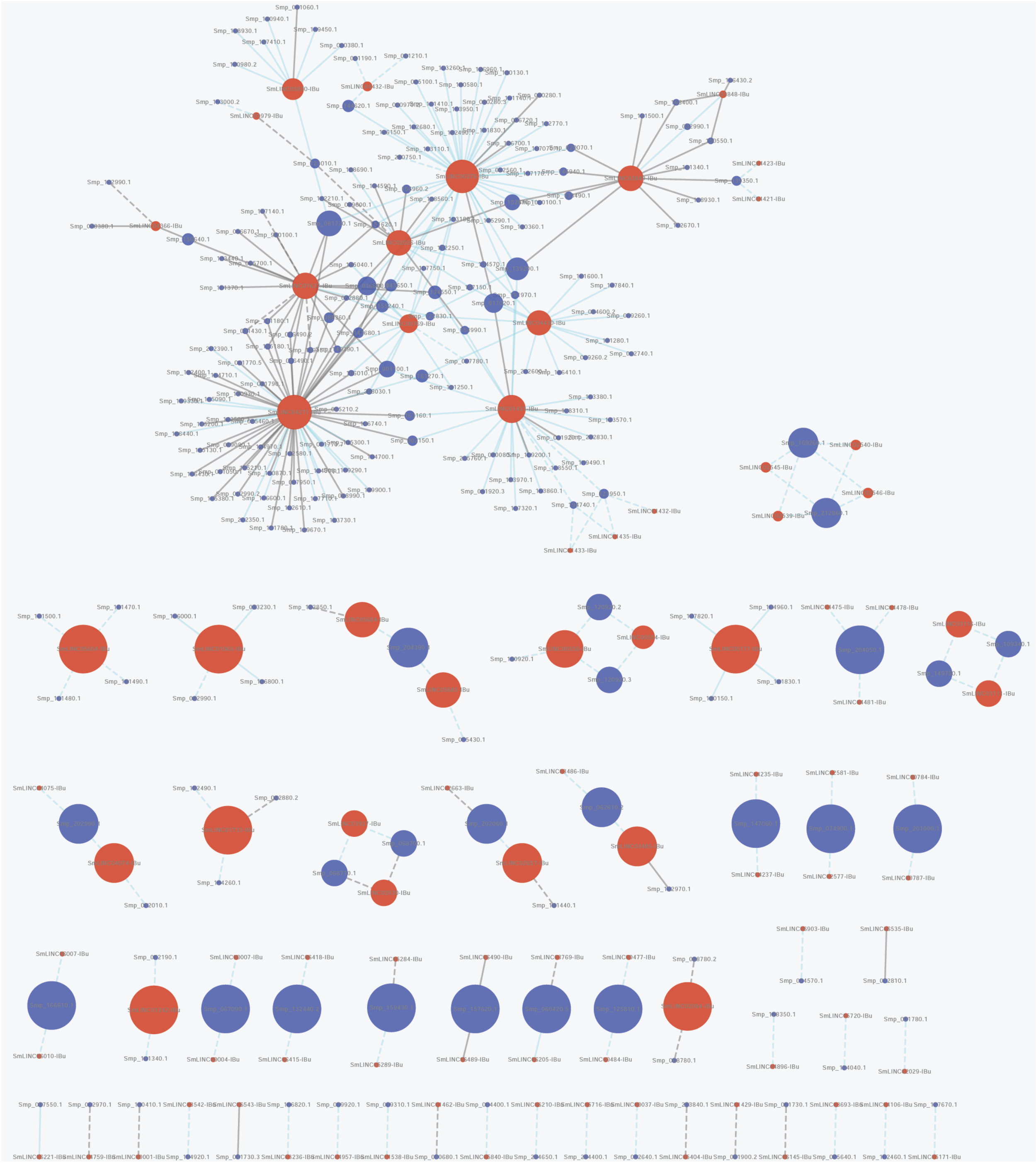

Figure S3. Complete view of the lincRNA-PC gene co-expression network built by the Topological Filtering Step (TFS) approach. This is the entire network from Fig 7A. The “Network Analyzer” function from cytoscape was used in order to obtain betweenness centrality score for each node, meaning the bigger the node, the higher propensity to be a hub. The network contains 89 SmLINC nodes (red) and 237 PC gene (Smp) nodes (blue), with 62.5% of positively correlated pairs (204 / 326, $r \geq 0.5$, cyan edges) and 37.5% of negatively correlated ones (122 / 326, $r \leq -0.5$, gray edges). Dashed edges represent neighbor genes.

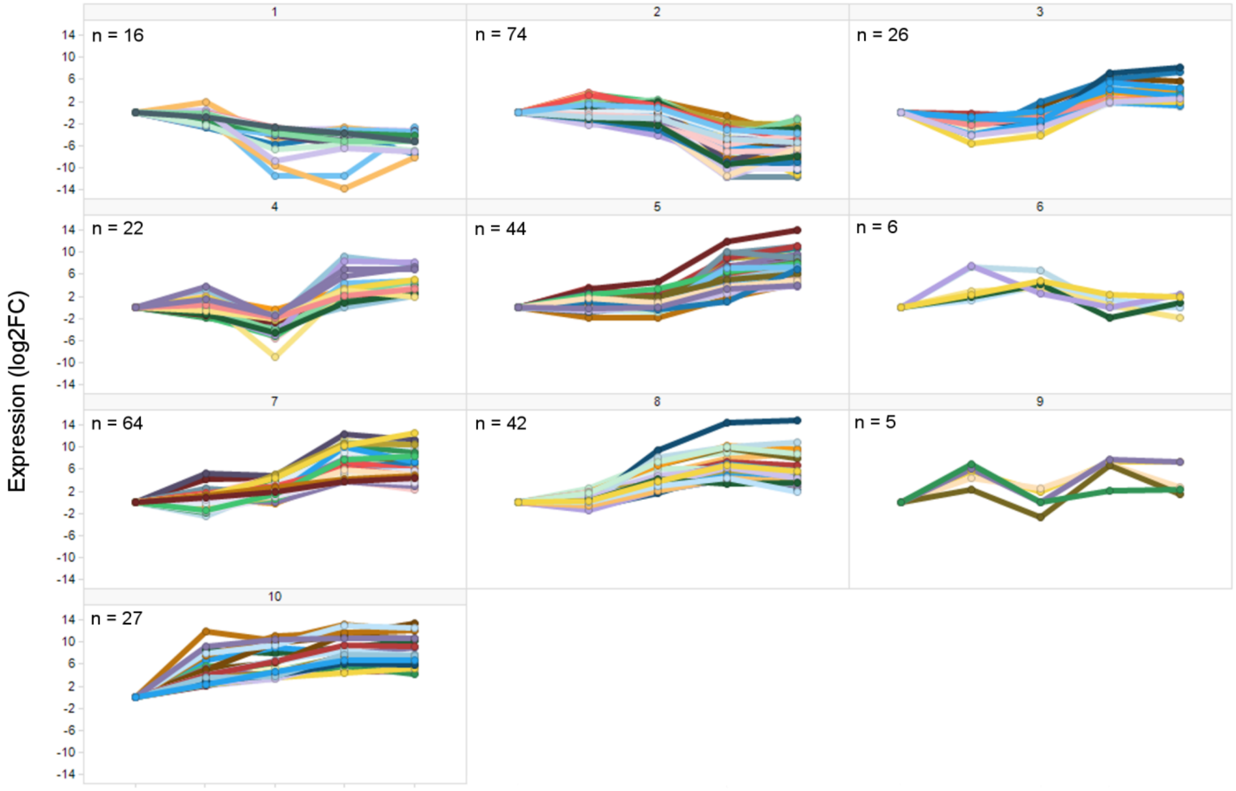

Figure S4. K-means analysis on all the 326 genes present in the entire network from Fig C. We performed the K-means analysis with 10 sets, being able to detect that 230/326 (70%) of the genes were grouped into clusters that displayed an up-regulation pattern from cercariae to adult forms (clusters 3, 4, 5, 7, 8, 9 and 10).

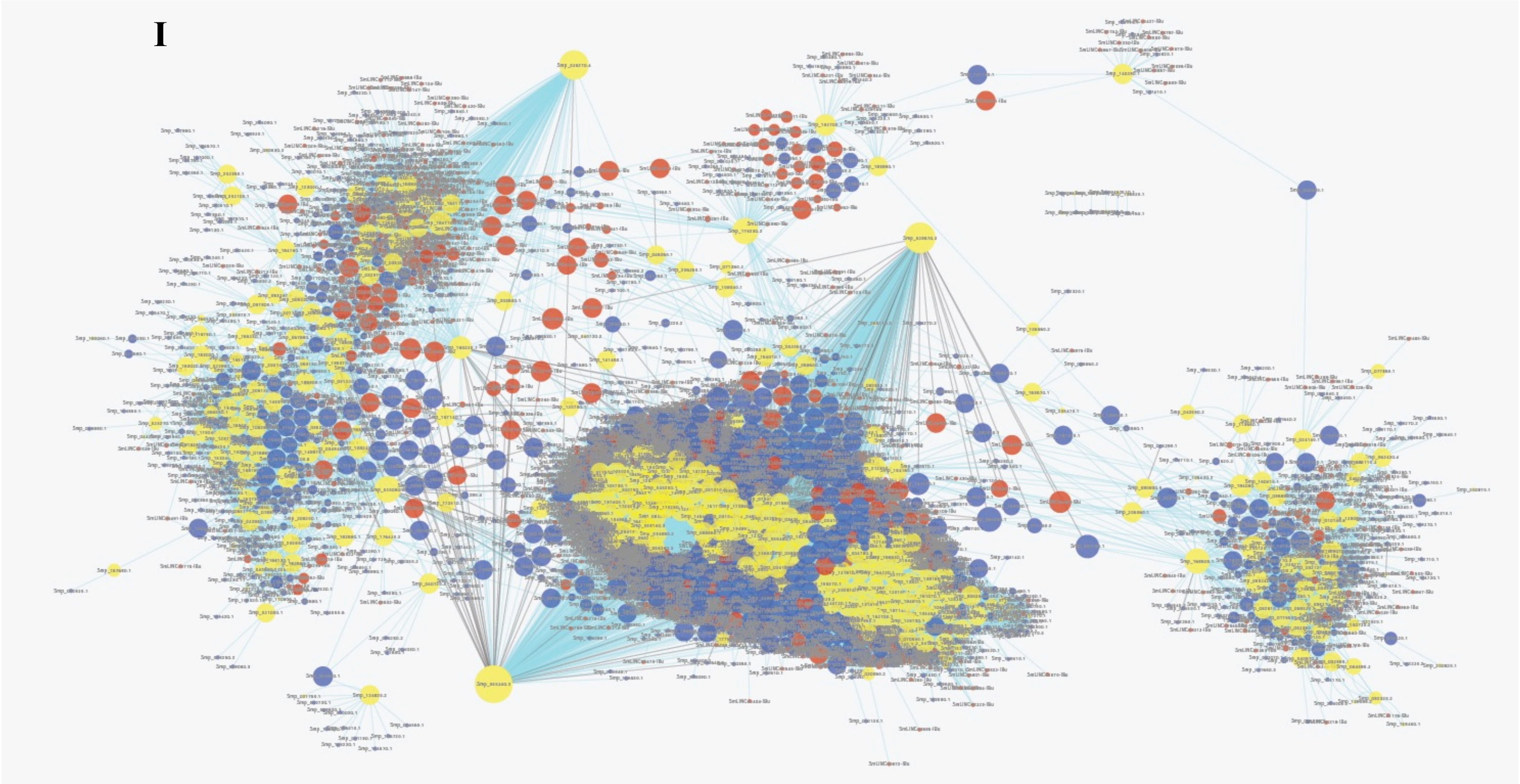

Figure S5. A second co-expression network construction approach highlights PC gene hubs and their correlated lincRNAs. (I) Network with 2965 nodes, 750 lincRNAs (red) + 2215 PC genes (blue and yellow), and 52,913 edges, of which 51,807 are for positive *r* (cyan) and 1106 for negative *r* (gray). The network was built by using 307 (yellow nodes) highly differentially expressed Smps ($log_2FC \geq 10$ on at least one out of five developmental stages) as bait in order to rescue all the other genes that presented a very stringent expression correlation coefficient ($r \geq 0.9$ or $r \leq -0.9$) with them.

II

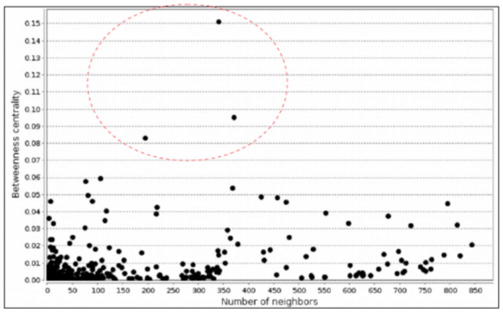

III

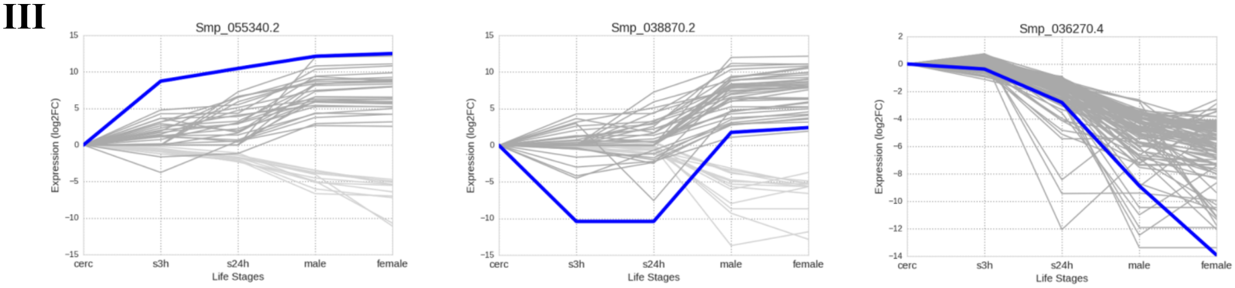

IV

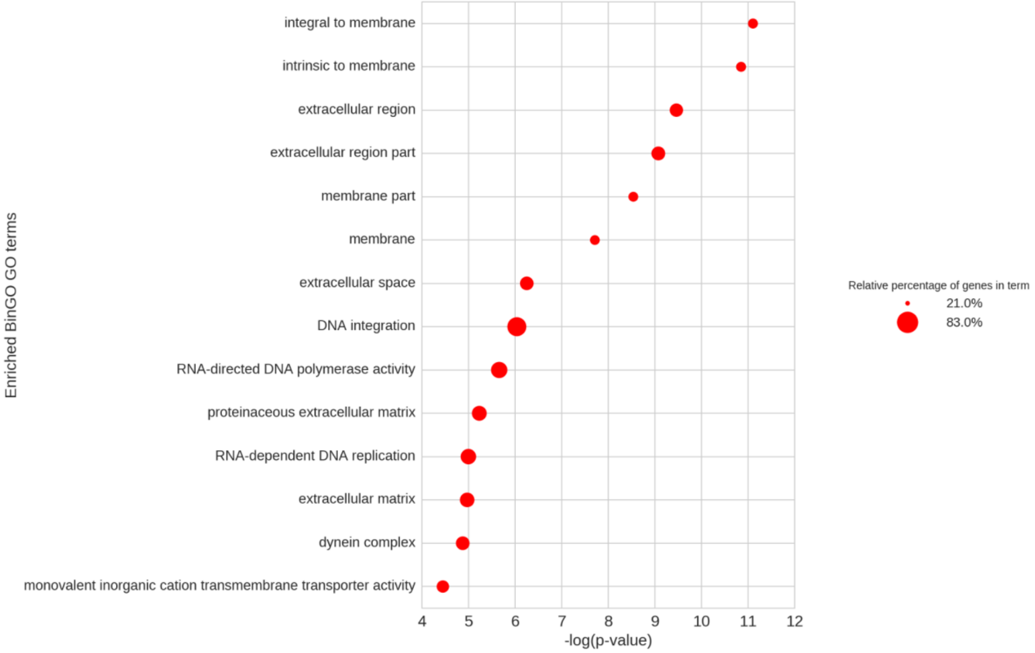

Figure S5. A second co-expression network construction approach highlights PC gene hubs and their correlated lincRNAs. (II) Betweenness centrality score calculation revealed three PC genes as the most important nodes in the network: Smp_055340.2, Smp_038870.2 and Smp_036270.4. **(III)** Co-expression kinetics on each subcluster from the three Smps described above (blue lines), displaying both positively (dark gray lines) and negatively (light gray lines) correlated SmLINCs across five life cycle stages: cercariae (cerc), somula 3h (s3h), somula 24h (s24h), male and female. **(IV)** Gene enrichment analysis on the 2215 PC genes in the network revealed significant GO terms, some of them identical to the ones depicted on Fig 6B from the 181 robust lincRNAs' network.

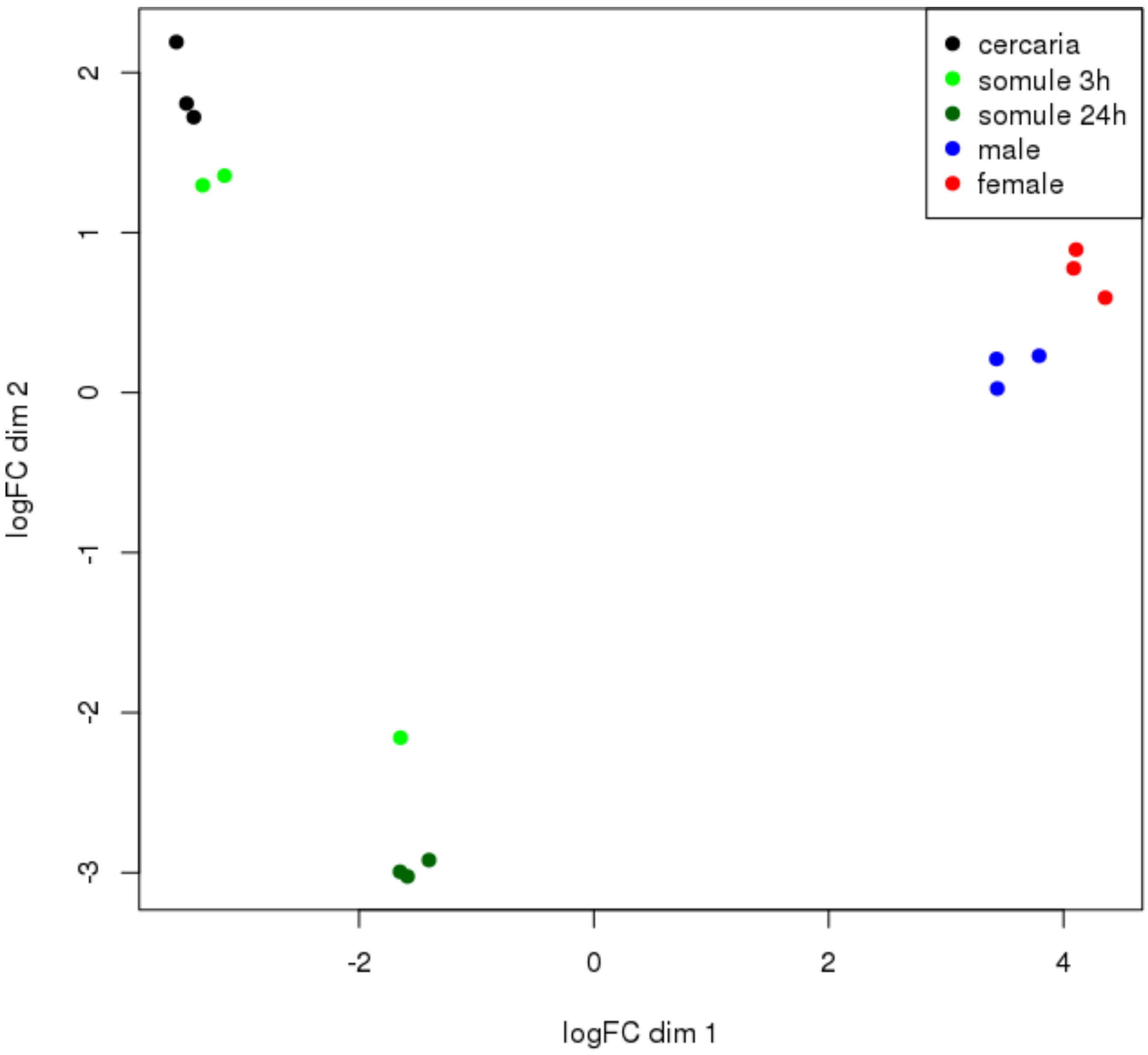

Figure S6. EdgeR multi-dimensional scaling plot (PCA-like) on five different developmental stages comprising 15 RNA-Seq samples of *S. mansoni* (biological triplicates for each stage). Cercariae (ERR022872, ERR022877 and ERR022878), somula 3h (ERR022874, ERR022876 and ERR022879), somula 24h (ERR022880, ERR022881 and ERR022882), male (SAMN06221530, SAMN06221531 and SAMN06221532) and female (SAMN06221542, SAMN06221543 and SAMN06221544) adults.

Supplementary References

1 Grabherr, M. G. *et al.* Full-length transcriptome assembly from RNA-Seq data without a reference genome. *Nat Biotechnol* **29**, 644-652, doi:10.1038/nbt.1883 (2011).
2 Heimburg, T. *et al.* Structure-Based Design and Synthesis of Novel Inhibitors Targeting HDAC8 from *Schistosoma mansoni* for the Treatment of Schistosomiasis. *J Med Chem* **59**, 2423-2435 (2016).
3 Verma, S. K. *et al.* Identification of Potent, Selective, Cell-Active Inhibitors of the Histone Lysine Methyltransferase EZH2. *ACS Med Chem Lett* **3**, 1091-1096 (2012).
4 Kim, D. *et al.* TopHat2: accurate alignment of transcriptomes in the presence of insertions, deletions and gene fusions. *Genome Biol* **14**, R36, doi:10.1186/gb-2013-14-4-r36 (2013).
5 Trapnell, C. *et al.* Transcript assembly and quantification by RNA-Seq reveals unannotated transcripts and isoform switching during cell differentiation. *Nat Biotechnol* **28**, 511-515, doi:10.1038/nbt.1621 (2010).
6 Bolger, A. M., Lohse, M. & Usadel, B. Trimmomatic: a flexible trimmer for Illumina sequence data. *Bioinformatics* **30**, 2114-2120 (2014).
7 Kent, W. J. BLAT--the BLAST-like alignment tool. *Genome Res* **12**, 656-664 (2002).
8 Quinlan, A. R. & Hall, I. M. BEDTools: a flexible suite of utilities for comparing genomic features. *Bioinformatics* **26**, 841-842 (2010).
9 Schmieder, R., Lim, Y. W. & Edwards, R. Identification and removal of ribosomal RNA sequences from metatranscriptomes. *Bioinformatics* **28**, 433-435 (2012).
10 Rice, P., Longden, I. & Bleasby, A. EMBOSS: the European Molecular Biology Open Software Suite. *Trends Genet* **16**, 276-277 (2000).
11 Kong, L. *et al.* CPC: assess the protein-coding potential of transcripts using sequence features and support vector machine. *Nucleic Acids Res* **35**, W345-349 (2007).
12 Jones, P. *et al.* InterProScan 5: genome-scale protein function classification. *Bioinformatics* **30**, 1236-1240 (2014).
13 Lepesant, J. M., Boissier, J., Climent, D., Cosseau, C. & Grunau, C. Female biased sex-ratio in *Schistosoma mansoni* after exposure to an allopatric intermediate host strain of *Biomphalaria glabrata*. *Exp Parasitol* **135**, 350-356 (2013).
14 Roquis, D. *et al.* Exposure to hycanthone alters chromatin structure around specific gene functions and specific repeats in *Schistosoma mansoni*. *Front Genet* **5**, 207 (2014).
15 Roquis, D. *et al.* The Epigenome of *Schistosoma mansoni* Provides Insight about How Cercariae Poise Transcription until Infection. *PLoS Negl Trop Dis* **9**, e0003853 (2015).
16 Anderson, L. *et al.* *Schistosoma mansoni* Egg, Adult Male and Female Comparative Gene Expression Analysis and Identification of Novel Genes by RNA-Seq. *PLoS Negl Trop Dis* **9**, e0004334, doi:10.1371/journal.pntd.0004334 (2015).
17 Heinz, S. *et al.* Simple combinations of lineage-determining transcription factors prime cis-regulatory elements required for macrophage and B cell identities. *Molecular cell* **38**, 576-589, doi:10.1016/j.molcel.2010.05.004 (2010).
18 Schwartz, S. *et al.* Human-mouse alignments with BLASTZ. *Genome Res* **13**, 103-107 (2003).
19 Blanchette, M. *et al.* Aligning multiple genomic sequences with the threaded blockset aligner. *Genome Res* **14**, 708-715, doi:10.1101/gr.1933104 (2004).

20 Siepel, A. *et al.* Evolutionarily conserved elements in vertebrate, insect, worm, and yeast genomes. *Genome Res* **15**, 1034-1050 (2005).
21 Siepel, A. & Haussler, D. Phylogenetic estimation of context-dependent substitution rates by maximum likelihood. *Mol Biol Evol* **21**, 468-488 (2004).
22 Bray, N. L., Pimentel, H., Melsted, P. & Pachter, L. Near-optimal probabilistic RNA-seq quantification. *Nat Biotechnol* **34**, 525-527 (2016).
23 Robinson, M. D., McCarthy, D. J. & Smyth, G. K. edgeR: a Bioconductor package for differential expression analysis of digital gene expression data. *Bioinformatics* **26**, 139-140 (2010).
24 Shannon, P. *et al.* Cytoscape: a software environment for integrated models of biomolecular interaction networks. *Genome Res* **13**, 2498-2504 (2003).
25 Buske, F. A., Bauer, D. C., Mattick, J. S. & Bailey, T. L. Triplexator: detecting nucleic acid triple helices in genomic and transcriptomic data. *Genome Res* **22**, 1372-1381 (2012).
26 Zhang, J. D. & Wiemann, S. KEGGgraph: a graph approach to KEGG PATHWAY in R and bioconductor. *Bioinformatics* **25**, 1470-1471 (2009).
27 Necsulea, A. *et al.* The evolution of lncRNA repertoires and expression patterns in tetrapods. *Nature* **505**, 635-640 (2014).
28 Maere, S., Heymans, K. & Kuiper, M. BiNGO: a Cytoscape plugin to assess overrepresentation of gene ontology categories in biological networks. *Bioinformatics* **21**, 3448-3449 (2005).
29 Basch, P. F. Cultivation of Schistosoma mansoni in vitro. I. Establishment of cultures from cercariae and development until pairing. *J Parasitol* **67**, 179-185 (1981).
30 Parker-Manuel, S. J., Ivens, A. C., Dillon, G. P. & Wilson, R. A. Gene expression patterns in larval *Schistosoma mansoni* associated with infection of the mammalian host. *PLoS Negl Trop Dis* **5**, e1274 (2011).
